# Temporal confounds emulate multivariate fMRI measures of perceptual learning

**DOI:** 10.1162/IMAG.a.1435

**Published:** 2026-11-04

**Authors:** Georgia A. Milne, Kim Staeubli, Hugo T. Chow-Wing-Bom, John A. Greenwood, Peter Kok, Roni O. Maimon-Mor, Tessa M. Dekker

**Affiliations:** Institute of Ophthalmology, University College London, London, United Kingdom; Experimental Psychology, Division of Psychology and Language Sciences, University College London, London, United Kingdom; Department of Imaging Neuroscience, UCL Queen Square Institute of Neurology, University College London, London, United Kingdom

**Keywords:** fMRI, RSA, perception, perceptual reorganisation, two-tone images, cognition

## Abstract

Any study of learning must capture neural or behavioural changes over time. Stimuli are therefore presented more than once, before and after learning takes place—a sequence that cannot be randomised across time. This design creates vulnerability to temporal confounds that cause illusory neural effects. We investigate this issue in a classic ‘one-shot’ learning paradigm, in which ambiguous two-tone images become recognisable after viewing a photo cue. Across common fMRI designs, we found that spurious correlations driven by the temporal proximity of stimuli created strong artefacts in multivariate data. Among certain experimental designs, we found strong neural markers of perceptual learning depending on how stimuli were distributed across these artefacts. This paradigm-dependent effect was present not only in the primary visual cortex (V1), but also in a non-visual control region (S1), indicative of an artefactual origin rather than a genuine perceptual learning effect. We present a principled approach to correct for this temporal order confound. Once applied, the artefactual learning signature was eliminated from both V1 and S1, and while the corrected signal retained information about image identity in V1, this was absent in S1. Our work demonstrates how current multivariate neuroimaging approaches to understanding learning could be influenced by methodological artefacts that misdirect interpretations towards distributed neural processing, and offers a practical framework to distinguish true learning from measurement error.

## 1 Introduction

Sensory representations are constantly updated as we learn. For example, long-term exposure to a particular species of animal can enhance their subtle features, while hearing a familiar voice can make you instantly recognise someone you had assumed was a stranger. In cognitive neuroscience, these perceptual dynamics are often studied with cueing tasks (Carrasco, 2018; Schacter & Buckner, 1998), in which cues that manipulate knowledge or attention can alter neural responses to subsequent stimuli. With the emergence of multivariate fMRI techniques, this is typically measured via representational similarity analysis (RSA), where variations in neural representations to different stimuli are assessed by correlating their multi-voxel responses (Kriegeskorte et al., 2008). While these measures are informative and robust to various univariate signal fluctuations (Kriegeskorte et al., 2008; Roth & Merriam, 2023), structured noise in the temporal domain can affect their results (Alink et al., 2015; Cai et al., 2019; Mumford et al., 2014). This could prove particularly problematic for learning paradigms, as they often require stimuli to be presented in a specific order (e.g., pre-cueing target, cue, post-cueing target), making full randomisation or counterbalancing impossible in the temporal domain.

A popular cueing task uses two-tone images to study perceptual reorganisation, or ‘one-shot’ learning (Armendariz et al., 2025; Chang et al., 2016; Davies et al., 2018; Dolan et al., 1997; Flounders et al., 2019; González-García et al., 2018; González-García & He, 2021; Hsieh et al., 2010; Lazarova et al., 2025; Ludmer et al., 2011; Teufel et al., 2015, 2018; van Loon et al., 2016; Yoon et al., 2007). In this phenomenon, an unrecognisable two-tone image, created by smoothing and contrast-binarising a greyscale photo (see Fig. 1A; Mooney, 1957), becomes recognisable only after exposure to the original photo. Perceptual reorganisation can therefore be robustly triggered by the sequential presentation of a two-tone image, its corresponding photo cue, and the same two-tone again (Ramachandran, 1994). Because the newly acquired knowledge can provoke distinct perceptual experiences in response to two presentations of the same stimulus, two-tone paradigms offer a well-controlled approach for measuring top-down modulation of sensory inputs across the brain.

**Fig. 1.**
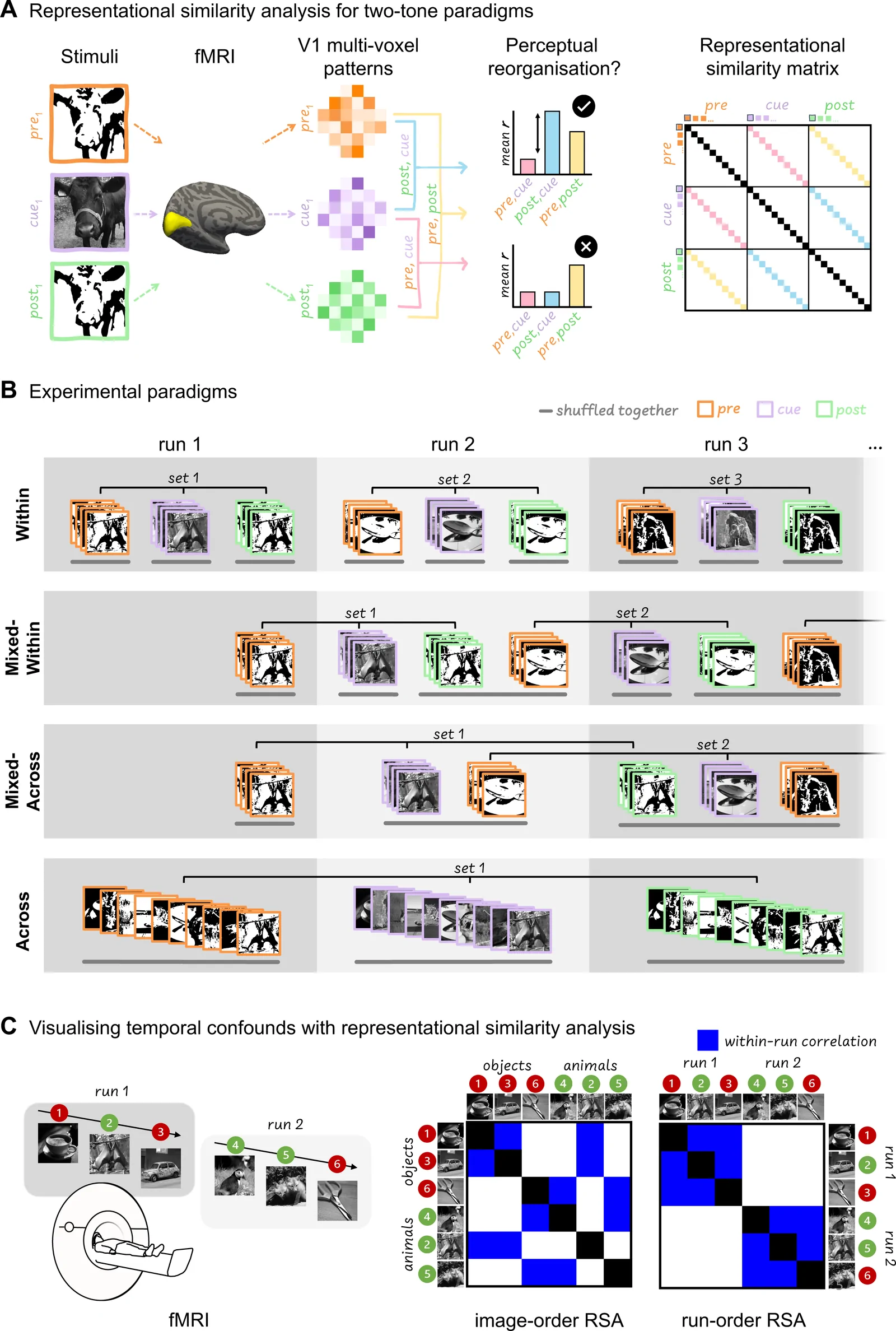
Perceptual reorganisation paradigms and analyses. (A) Experimental procedure and representational similarity matrix (RSM) construction for measuring perceptual reorganisation with fMRI. (B) Paradigm sequences of the four experimental designs tested shown for the first three fMRI runs of each design (total number of runs: Within, five runs; Mixed-Within, six runs; Mixed-Across, seven runs; Across, six runs). Each image is shown in ‘Pre’ condition (orange borders), ‘Cue’ condition (lilac borders) and ‘Post’ condition (green borders). Groups of images shuffled together are underlined with grey bars. (C) Temporally driven pattern correlations, for example, positive correlations of trials within the same run (within-run correlations), are obscured by conventional RSA methods that order data according to image identity (image-order RSA, left matrix), but can be revealed by ordering the same data by the sequence in which stimuli were presented across the experiment (run-order RSA, right matrix).

RSA can reveal whether two-tone images are represented more similarly to their photo versions after cueing, in line with the phenomenological experience of perceptual reorganisation (Fig. 1A). Hsieh and colleagues were the first to identify this neural marker of perceptual reorganisation (Hsieh et al., 2010). They observed increased correlations between photo and two-tone stimuli following cueing in both the Lateral Occipital Cortex (LOC)—a higher-order area known to play a critical role in object recognition (Grill-Spector et al., 2000; Malach et al., 1995)—and in the early visual cortex, suggesting widespread knowledge-based modulation of neural representations (Hsieh et al., 2010). Van Loon and colleagues later extended these findings, observing perceptual reorganisation effects from the primary visual cortex (V1) to the posterior fusiform (pFs), including intermediate processing stages. These were diminished in lower-order areas when NMDA-mediated feedback processing was pharmacologically inhibited (Van Loon et al., 2016). More recently, González-García and colleagues replicated findings of distributed knowledge-driven processing, and further identified classically ‘non-visual’ areas, such as the frontoparietal (FPN) and default-mode (DMN) networks, as encoders of perceptual reorganisation (González-García et al., 2018; González-García & He, 2021). Strikingly, these studies find that early visual areas represent cued two-tones more similarly to their photo versions than to the same two-tones viewed before cueing (González-García et al., 2018; Hsieh et al., 2010; van Loon et al., 2016). While top-down processing has previously been found to modulate representations throughout the visual hierarchy in other perceptual tasks (Kok et al., 2012, 2016; Lamme & Roelfsema, 2000; Poltoratski & Tong, 2020; Self et al., 2013; Yon et al., 2023), the findings from perceptual reorganisation studies extend this considerably by reporting effects of prior knowledge that are widespread across the cortex, and that can dominate incoming visual input.

The above findings offer compelling insights into long-standing neuroscientific debates about the distribution of conscious perceptual processing, leveraging a range of paradigm designs to reach corroborating conclusions about the strength and distribution of top-down neural signals. However, the designs used share a feature that is common to all perceptual reorganisation paradigms: uncued two-tones (‘Pre’ condition) must always precede their corresponding photo versions (‘Cue’ condition), which, in turn, must always precede the corresponding cued two-tones (‘Post’ condition, Fig. 1A). As this presentation order is necessary for triggering and measuring perceptual reorganisation, it results in a temporal consistency across subjects that cannot be avoided by trial randomisation or counterbalancing. This raises questions about the potential influence of temporal artefacts on RSA measures of perceptual reorganisation, which may be overlooked when using conventional methods of reporting these data (Fig. 1C).

In fMRI, structured noise can arise from both physiological and methodological sources, and can act across multiple temporal levels. For example, the subject’s motion, breathing, heart-rate and attentional drift, alongside thermal fluctuations, scanner instability, equipment radio frequency signals, and preprocessing and analysis artefacts can alter neural signals between scanning sessions, within a single session, between fMRI runs, and within single runs (Aguirre et al., 1997; Alink et al., 2015; Cai et al., 2019; Mumford et al., 2014). The combined impact of structured noise on multi-voxel patterns may therefore be complex, and variable across subjects, set-ups, and paradigm designs. To investigate this issue, we varied how stimuli were temporally distributed in four fMRI two-tone paradigms, including established designs used in the literature, and characterised the impacts of temporal effects at the single-subject level in each design. The paradigms differed in how stimulus conditions (Pre, Cue and Post) were arranged within and across fMRI runs, and should therefore vary in their sensitivity to structured noise across different temporal levels. In an across-run design (Gorlin et al., 2012; Hsieh et al., 2010; Lazarova et al., 2025; van Loon et al., 2016), separate fMRI runs are dedicated to each stimulus condition. This allows trials to be fully randomised within each run without disrupting the required order of conditions, but results in long delays between presentations of corresponding stimuli (‘Across’ design; Fig. 1B, bottom row). In a ‘Mixed-Within’ design (Flounders et al., 2019; González-García et al., 2018; González-García & He, 2021), Pre condition two-tones are presented in a separate run to the corresponding Cue and Post conditions, which are shown in the following run alongside new Pre images. This design counterbalances stimulus conditions more evenly across the experiment, but unsymmetrically across fMRI runs (Fig. 1B, second row). In a Within-run design (Hegde & Kersten, 2010; Ludmer et al., 2011), all conditions of a set of images are presented within a single run, meaning trials can only be randomised within subsections of each run, but effect sizes may be enhanced by avoiding across-run signal variability and minimising delays between presentations of corresponding stimuli (Fig. 1B, first row). We also included a ‘Mixed-Across’ design to offer a logical alternative for managing temporal order constraints while counterbalancing stimulus conditions. Here, a single run can contain all three stimulus conditions, but Pre, Cue, and Post conditions of the same images are always separated across three consecutive runs, allowing for full randomisation of all images presented within a run (Fig. 1B, third row).

For each of the above four designs, we presented the same 20 images of natural and man-made scenes, and measured perceptual reorganisation behaviourally and cortically at the single-subject level. Our cortical regions of interest included primary visual cortex (V1) and primary somatosensory cortex (S1) as a control region. While previous studies have reported strong patterns of perceptual reorganisation in V1 (González-García et al., 2018; González-García & He, 2021; van Loon et al., 2016), S1, as a typically non-visual region, should not be sensitive to perceptual reorganisation of visual stimuli, but may show design-driven artefacts. Using conventional RSA measures, we find that some paradigms result in strong neural patterns of perceptual reorganisation across both V1 and S1, while others do not. This leads us to identify a substantial temporal artefact present in all paradigms tested, that confounds conventional measures of perceptual reorganisation depending on the distribution of stimulus conditions across fMRI runs. We further show how even in confounded paradigms, appropriate control analyses can mitigate these artefactually enhanced effects. Critically, without these corrections, the true extent of temporal confounds can be masked by conventional RSA practices (Fig. 1C), yet can emulate strong neural patterns of perceptual reorganisation across the cortex. Based on this example from perceptual reorganisation, we recommend three strategies for distinguishing genuine learning effects from temporal artefacts in multivariate fMRI studies.

## 2 Methods

### 2.1 Participants

Nineteen healthy adults participated in the study. All participants had normal or corrected-to-normal vision and provided written informed consent before participating. Three participants were excluded: two due to poor task compliance and one due to a technical issue. Of the remaining 16 participants, 7 were female, 1 was left-handed, and the mean age was 26.3 years (age range: 19.8–30.9 years). The research was carried out in accordance with the tenets of the Declaration of Helsinki and was approved by the UCL Research Ethics Committee (project number 22397/001).

### 2.2 Stimuli

Stimuli were generated from 20 images of animals and objects from The Berkeley Segmentation Dataset and Benchmark (Martin et al., 2001) and the Open Images Dataset V7 (Kuznetsova et al., 2020) using MATLAB R2022a (The MathWorks Inc., 2022). Images were converted to greyscale and dynamically cropped to a square to create photo cues. Two-tone stimuli were created using methods previously described elsewhere (see Milne et al., 2024). Specifically, photo cues were smoothed with a 2D Gaussian filter and then thresholded to binarise pixel luminance to black or white, creating images with obscured edges. Smoothing levels and binarisation thresholds varied per image; standard deviation of Gaussian distribution ranged from 0.5–6.0 SD, binarisation thresholds ranged from 0.4–2.0 times the mean luminance of the greyscale image. An additional four two-tones generated with no smoothing were used in a practice task to test task comprehension. Five ‘Abstract’ two-tone images were also created by scrambling two-tone images, producing contentless two-tones for which photo cues do not exist, but are not used in analyses presented in this study. All stimuli were presented as a 13 degrees of visual angle square centred on a 50% grey background, and projected onto a screen at the back of the MRI scanner, which was viewed via a mirror.

### 2.3 Procedure

During an experimental trial, participants viewed a two-tone or a greyscale image and judged whether the presented image was of an animal or a man-made object. Verbal recognition of the specific item in the image was recorded after each scan in a verbal recognition task.

Each of the 16 participants completed one of four different task paradigms (see Fig. 1B): Within (n = 4), Mixed-Within (n = 4), Mixed-Across (n = 4), and Across (n = 4). This between-subjects design is necessary for comparing one-shot learning paradigms, as behavioural effects would diminish if the task were to be repeated within subjects. All paradigms included 20 images shown in three conditions: ‘Pre’ two-tone condition, ‘Cue’ greyscale photo condition, and ‘Post’ two-tone condition. In each paradigm, images and conditions were distributed across fMRI runs in different ways (see below). The order of presented images was shuffled per participant, to allow for maximal randomisation while maintaining the sequential constraints of the task (see Fig. 1B). Between fMRI runs, participants completed a verbal recognition task of the two-tone images to measure their pre- and post-cueing recognition.

The single-trial structure of all fMRI paradigms was identical. Each trial started with a black fixation cross presented for 1 s, followed by a 0.2 s stimulus presentation of either a photo cue or a two-tone image. This was followed by a grey fixation cross presented for 1.8 s, prompting the participant to respond via button press to whether the image contained an animal or an object. Each trial was followed by a variable inter-stimulus interval of 0.1 to 4.1 s, consisting of a grey fixation cross. In the verbal recognition task, participants saw each image for 0.2 s, and verbally answered the question ‘Can you name what you saw?’. Participants’ answers were typed on the participant’s screen by the experimenter, and corrected by the participant where necessary.

Before the fMRI experiment, participants first received task instructions and completed a short practice task outside of the scanner. This task comprised eight trials of four pairs of unsmoothed two-tones and corresponding photo cues, and was repeated until the participant correctly categorised all trials as either an animal or an object.

### 2.4 Within design

In the Within paradigm, each fMRI run included all three conditions of four images—first as a two-tone presented before cueing (‘Pre’ condition), second as a photo cue (‘Cue’ condition), and third as a two-tone presented after cueing (‘Post’ condition). In addition, one abstract two-tone image was shown alongside Pre and Post conditions for each run, but not as a photo cue. Each image was repeated six times with a total of 84 trials per run. Presentation order was pseudorandomised across images within the same condition, but not across conditions. To distribute the image repeats, we shuffled image orders of each condition within 3 trial blocks including 2 repeats of each image, which were pseudorandomised ensuring no image was repeated twice in a row. Three rest periods of 13 s duration were included at the start, middle (between Pre and Cue conditions), and end of each run, in which a grey fixation cross was shown. In total, the paradigm included five fMRI runs that each lasted 7 min 47 s. The verbal recognition task was completed between each run for the four two-tone images shown in the previous run (post-cueing recognition) and the four two-tones to be shown in the next run (pre-cueing recognition), and at the start and end of the experiment to test pre-cueing recognition of the first four two-tones, and post-cueing recognition of the last four two-tones, respectively.

### 2.5 Mixed-within design

In the Mixed-Within paradigm, fMRI runs included four images presented in Pre condition, and four different images presented in both Cue and Post conditions. In addition, one abstract image was shown alongside Pre and Post conditions for each run, but not as a photo cue. Images presented in Pre and Post conditions were repeated six times, and images presented in Cue condition were repeated three times. In total, there were 72 trials per run when all conditions were included (see below). Presentation order was pseudorandomised across the interspersed Pre and Post conditions—which contained different images. In the Cue condition, randomisation of trial order was carried out across images but not mixed with the other conditions. Trials were divided into two types of blocks: two-tone blocks, in which Pre, Post, and Abstract were combined, and Cue blocks. Each two-tone block included two repetitions of ten images in a pseudorandomised order. Cue blocks included only one repetition of four images in a pseudorandomised order. Both blocks were repeated 3 times, and Cue and two-tone blocks were interleaved, so a Cue block was always followed by a two-tone block. Three rest periods of 10 s duration were included at the start, middle (between two-tone and Cue conditions), and end of each run. The nature of this paradigm meant that we could not start with a run that included all three conditions, for example, to have images to show in the Cue and Post conditions they must first be presented in a separate run as Pre condition. Therefore, the paradigm included six fMRI runs of different lengths: run 1 included only the Pre condition (2 m 55 s), runs 2–5 included all conditions (6 m 35 s), and run 6 included only Cue and Post conditions (3 m 55 s). The verbal recognition task was completed after each run for any two-tone images shown in the previous run (pre- and post-cueing recognition).

### 2.6 Mixed-across design

In the Mixed-Across paradigm, each condition of corresponding images was presented in a separate run. fMRI runs included four images presented in Pre condition, four different images presented in Cue condition, and four different images presented in Post condition. In addition, one abstract image was shown alongside Pre and Post conditions for each run, but not as a photo cue. Images in Pre and Post conditions were repeated six times, and images presented in Cue condition were repeated three times. In total, there were 72 trials per run when all conditions were included (see below). Because all conditions within a run were of different images, presentation order was pseudorandomised across all trials (i.e., across images and conditions). To distribute the image repeats across the run, we shuffled image orders within 3 trial blocks including 2 repeats of the 4 Pre, and 4 Post two-tones, and 1 repeat of the 4 photo cues. Three rest periods of 10 s duration were included at the start, middle (between two-tone and ‘Cue’ conditions), and end of each run, in which a grey fixation cross was shown. The nature of this paradigm meant that we could not start with a run that includes all three conditions, for example, to have images to show in the Cue condition they must first be presented in a separate run in Pre condition. Therefore, the paradigm included seven fMRI runs of different lengths: run 1 included only the Pre condition (2 m 52 s), run 2 included Pre and Cue conditions (4 m 3 s), runs 3–5 included all conditions (6 m 33 s), run 6 included Cue and Post conditions (4 m 3 s), and run 7 included only Post condition (2 m 52 s). The verbal recognition task was completed after each run for any two-tone images shown in the previous run (pre- and post-cueing recognition).

### 2.7 Across design

In the Across paradigm, each condition (Pre, Cue or Post) was presented in a separate run. Each fMRI run included 10 images (half the stimulus set randomly split). In addition, two abstract images were shown in Pre and Post condition runs, but not in Cue runs. Each image was repeated six times, for a total of 72 trials per Pre/Post runs, and 60 trials per Cue run. To distribute the image repeats across the run, we shuffled image orders within 3 trial blocks including 2 repeats of each image. Three rest periods of 13 s duration were included at the start, middle, and end of each run, in which a grey fixation cross was shown. In total, the paradigm included two Pre condition runs (runs 1 and 4) that lasted 6 m 46 s each, two Cue condition runs (runs 2 and 5) that lasted 5 m 45 s each, and two Post condition runs (runs 3 and 6) that lasted 6 m 46 s each. The verbal recognition task was completed after each run that included two-tone images (runs 1, 3, 4, and 6) for the ten Pre or Post condition two-tone images shown in the previous run.

### 2.8 Data acquisition and preprocessing

Data were collected at the Birkbeck-UCL Centre for NeuroImaging (BUCNI, London, UK), on a Siemens PRISMA 3 Tesla scanner and a 30-channel head coil (identical to 32-channel but without view-obstructing front). T2*-weighted echo-planar imaging was collected with an accelerated multi-band sequence by CMRR (version R016a, www.cmrr.umn.edu/multiband; Cauley et al., 2014; Xu et al., 2013); multi-band factor: 4, voxel resolution: 2 mm isotropic, FOV: 212 × 212 × 96 mm, flip angle: 60°, repetition time (TR): 1000 ms, echo time (TE): 35.2 ms, echo spacing: 0.56 ms, bandwidth: 2620 Hz/Px, with 48 transverse slices angled to be approximately parallel to the calcarine sulcus whilst avoiding the orbital cavities.

A T1-weighted structural image was acquired with a 30-channel coil, using MPRAGE sequence (voxel resolution: 1 mm isotropic, 208 slices, FOV: 256 × 256 × 208 mm, flip angle: 9°, TR = 2.3 s, TE = 2.98 ms, TI = 900 ms, bandwidth: 240 Hz/Px, acquisition time: 5 min 30 s).

Preprocessing of structural and functional data was performed with FSL (FMRIB’s Software Library, www.fmrib.ox.ac.uk/fsl). Structural images were skull-stripped, and functional images were motion assessed using framewise displacement (threshold = 0.9). Functional data processing was carried out using FSL’s FEAT (FMRI Expert Analysis Tool) Version 6.00 (www.fmrib.ox.ac.uk/fsl), including: motion correction using MCFLIRT (Jenkinson et al., 2002), non-brain removal using BET (Smith, 2002), spatial smoothing using a Gaussian kernel of FWHM 3 mm, grand-mean intensity normalisation by a single multiplicative factor, and high-pass temporal filtering (Gaussian-weighted least-squares straight line fitting, with sigma = 50.0 s). Time-series statistical analysis was carried out using FILM with local autocorrelation correction (Woolrich et al., 2001). Specifically, a general linear model (GLM) was constructed at the single-subject level in native functional space. Regressors for each image (Im_1_ to Im_20_) in each condition (Pre, Cue and Post) that included all trial repeats of that stimulus, as well as regressors of suprathreshold movement confounds, were convolved with the hemodynamic response function (HRF) following a double gamma function. First-order temporal derivatives of each regressor were also included in the model. Functional images were registered to high-resolution structural scans using FLIRT (Jenkinson et al., 2002; Jenkinson & Smith, 2001) with boundary-based registration (BBR), and these high-resolution images were registered to a 2 × 2 × 2 mm MNI atlas using FNIRT nonlinear registration (Andersson et al., 2007) with 12 degrees of freedom.

### 2.9 Region of interest definition

Regions of interest (ROIs), primary visual cortex (V1) and primary somatosensory cortex (S1), were anatomically defined for each participant. For V1, cortical reconstruction was performed in FreeSurfer 7.3.2 (https://surfer.nmr.mgh.harvard.edu/fswiki/recon-all). A cortical V1 label was then obtained using Benson retinotopy (Benson & Winawer, 2018), which was restricted to an eccentricity of 9 degrees of visual angle to match the visually stimulated region. Restricted V1 labels were then converted to volumetric masks using FreeSurfer’s BBregister and combined across hemispheres. For S1, volumetric masks were anatomically defined in standard space using the Harvard-Oxford Cortical Structural Atlas (https://fsl.fmrib.ox.ac.uk/fsl/docs/other/datasets.html) template region for the postcentral gyrus, and registered back to the native space of each subject.

### 2.10 Representational similarity analysis

For each ROI (V1 and S1) the GLM beta-weights per run were extracted and normalised with multivariate noise normalisation (Walther et al., 2016) using the Representational Similarity Analysis Toolbox (Nili et al., 2014) adapted to FSL (www.zenodo.org/records/1112437) in MATLAB version R2024b (The MathWorks Inc., 2024). Pairwise comparisons between normalised multivariate patterns were performed using Pearson correlation as the distance measure. All pairwise comparisons were then visualised in a 60 x 60 Representational Similarity Matrix (RSM). For each subject, we constructed two RSMs, in which images were arranged along the rows and columns based on two factors: the condition in which the image appeared (i.e., rows 1–20 = Pre, rows 21–40 = Cue, and rows 41–60 = Post) and within each condition either (1) an arbitrary ordering of image identities (‘image-order’) or (2) the order in which the images were presented to each individual subject during scanning (‘run-order’). Specifically, the sequence by which run-order RSMs are arranged corresponds to which fMRI run a given condition of an image appeared. For example, Within, Mixed-Within and Mixed-Across paradigms present four unique images in Pre condition per run, so correlations of the Pre condition images presented in run 1 are displayed in run-order RSM rows 1–4, images presented in run 2 are displayed in rows 5–8, and so on. The same order applies across all three stimulus conditions, with subsequent conditions presented according to the paradigm design (see Fig. 1B). The order of images within these four-row blocks is arbitrary, as images are repeated multiple times within a run in blocks with different orders. Ordering RSMs by this order for each stimulus condition generates a matrix structure where images presented in the same run appear in consecutive rows within each condition block, while corresponding images across conditions can be identified along the off-centre diagonal sections of the RSM. Both image-order and run-order RSMs were also constructed and analysed using non-normalised beta values with similar results (see Supplementary Fig. S3).

### 2.11 Temporal controls

To control for temporal artefacts in across-condition comparisons of corresponding images, we computed temporal controls using non-corresponding images that had similar temporal presentation patterns. These captured the same temporal correlation structure but excluded content-specific information. A temporal control was calculated for each set of images that were grouped together in the experimental design (sets of 4 images in Within, Mixed-Within, and Mixed-Across paradigms; sets of 10 images for Across paradigm).

In the ‘Within’ paradigm, all corresponding image comparisons occur within the same run. To create temporal controls for Pre&Cue comparisons, for example, we calculated correlations between all non-corresponding Pre and Cue images within each run (e.g., for a 4-image set size, this yields 12 correlations: Pre image1 with Cue images 2, 3, & 4, Pre image2 with Cue images 1, 3, & 4, etc.). These correlations were averaged to create a run-specific temporal control value for the Pre&Cue comparison. To correct for temporal artefacts, each corresponding image correlation (e.g., Pre image1 & Cue image1) was corrected by subtracting the mean temporal control correlation taken from its respective run. This process was repeated for all across-condition comparisons (Pre&Cue, Post&Cue, Pre&Post).

In the Mixed-Within paradigm, the temporal structure differs, but the logic is the same. In this design, Pre images from one run are compared with their corresponding Cue and Post images from the subsequent run. For Pre&Cue temporal controls, we therefore calculated correlations between non-corresponding Pre and Cue images that maintained this across-run structure (e.g., Pre image1 in run 1 with Cue images 2, 3, & 4 in run 2). Similarly, Pre&Post temporal controls used non-corresponding images spanning these different runs. However, Post&Cue comparisons had a different structure: both Post and Cue images appeared within the same run, so temporal controls were calculated between non-corresponding Post and Cue images within that run (e.g., Post image1 with Cue images 2, 3, & 4, all in run 2).

In the Mixed-Across paradigm, all comparisons span different runs (e.g., Pre in run 1, Cue in run 2, Post in run 3), so temporal controls matched these specific across-run distances. For Pre&Cue comparisons, temporal controls were calculated using correlations between non-corresponding Pre and Cue images that maintained the same run separation (e.g., for a 4-image set, this yields 12 correlations: Pre image 1 in run 1 with Cue images 2, 3, & 4 in run 2, etc.). These correlations were averaged to create temporal control values that captured the across-run correlation structure. The same logic applied to Post&Cue and Pre&Post comparisons, with temporal controls always matching the specific run distances of the corresponding image comparisons.

The Across paradigm had a fundamentally different structure: each stimulus condition was presented in dedicated runs (runs 1 and 4 for Pre, runs 2 and 5 for Cue, runs 3 and 6 for Post). Additionally, the Across paradigm used larger image sets (10 images per set instead of 4), resulting in 90 non-corresponding correlations averaged for each run-specific temporal control (10 × 10 matrix excluding 10 diagonal pairs). To correct for temporal artefacts, for example, the correlation between Pre image 1 & Cue image 1 (where Pre image 1 was presented in run 1 and Cue image 1 in run 2) would be corrected by subtracting the temporal control value calculated from all non-corresponding correlations between Pre images in run 1 and Cue images in run 2 (e.g., Pre image 1 & Cue image 2, Pre image 2 & Cue image1, etc.).

### 2.12 Statistical analyses

All statistical analyses were carried out in MATLAB version R2024b, equipped with the Statistics and Machine Learning Toolbox and the Bioinformatics Toolbox (2024).

To assess perceptual reorganisation behaviourally, we modelled subjects’ behavioural responses in the image recognition and image categorisation tasks. For each paradigm, all trials for all participants were analysed using a generalised linear mixed model (function fitglme) with random intercepts for subjects and images on accuracy of image recognition responses (a binomial measure of image recognition recorded as either recognised or unrecognised once for each image in Pre and Post conditions), and a linear mixed model (function fitlme) with the same random intercepts on accuracy of image categorisation responses (for each image, an average of the six repeated image categorisation scores in Pre and Post conditions).

To assess neural perceptual reorganisation in each cortical ROI (V1 and S1), we calculated pairwise correlations between representations of corresponding images (within-image ***r***) in each stimulus condition (Pre&Cue, Post&Cue and Pre&Post) at the single-subject level. We then ran paired t-tests between the three across-condition comparisons of all 20 image pairs within each subject and ROI. Multiple comparison corrections were performed using FDR (see Supplementary Table S1).

To test for differences between within- and between-run correlations across all paradigms, we ran a linear mixed-effects model (function fitlme) of Fisher z-transformed across-condition correlations of V1 responses to corresponding images. The model included random intercepts for subjects and images, and tested for main effects of comparison type (within- or between-run), condition pair (Pre&Cue, Post&Cue or Pre&Post), and the interaction between these factors.

To test for differences in representational similarity across the conditions due to temporal correlation structure unrelated to stimulus identity, we ran paired t-tests between the three across-condition comparisons of all temporal controls, and ran FDR multiple comparisons corrections (see Supplementary Table S2).

To test for the isolated effects of temporal artefacts, and the underlying perceptual reorganisation effects after these have been removed, we ran paired t-tests between each across-condition comparison (Pre&Cue, Post&Cue, and Pre&Post) of all 20 image pairs within each subject and ROI for corrected within-image similarities, and ran FDR multiple comparisons corrections (see Supplementary Table S3).

## 3 Results

We assessed four fMRI paradigm designs of a two-tone cueing task that included 20 images presented in three conditions: as a naïvely viewed two-tone (Pre), followed by the original photo (Cue), and the same two-tone viewed after cueing (Post). The paradigms varied in how these conditions were organised across fMRI runs (Fig. 1B); with the three conditions of any given image presented in either a single run (‘Within’ paradigm), two runs (‘Mixed-Within’ paradigm), or three runs (‘Mixed-Across’ and ‘Across’ paradigms). For all paradigms, participants completed a verbal recognition task between fMRI runs, and an animate/inanimate categorisation task during each trial. Photo cueing substantially improved two-tone recognition, increasing the average number of correctly identified images from 34% to 91% across all designs (Supplementary Fig. S1). A generalised linear mixed model confirmed significant effects of two-tone condition on image recognition within each paradigm (β > 1.55, SE < 0.8, t > 4.07, *p* < 10^-4^; see Supplementary Text S1 for details).

We next tested for neural markers of perceptual reorganisation using representational similarity analysis (RSA; Kriegeskorte et al., 2008). After running a GLM, we used the univariate parameter estimates to calculate the average multi-voxel response pattern elicited by each of the 20 images (Im_1_ to Im_20_) for each of the three stimulus conditions (Pre, Cue, and Post). We restricted these patterns to our two cortical regions of interest (ROIs): primary visual cortex (V1), where substantial perceptual reorganisation has been previously reported, and a control region, primary somatosensory cortex (S1), which would not be expected to encode visual perception. Next, we applied multivariate noise normalisation (Walther et al., 2016) and compared all 60 response patterns to each other via pairwise correlations (*Pearson’s **r***). We first constructed image-order representational similarity matrices (RSMs), in which each square represents the similarity of multi-voxel responses to two individual image presentations (group-averaged RSMs shown in Fig. 2A, representative single-subject RSMs shown in Supplementary Fig. S2). To quantify measures of perceptual reorganisation, we calculated the mean similarity for each across-condition image pair in single-subject RSMs (within-image ***r***; Fig. 2A).

**Fig. 2.**
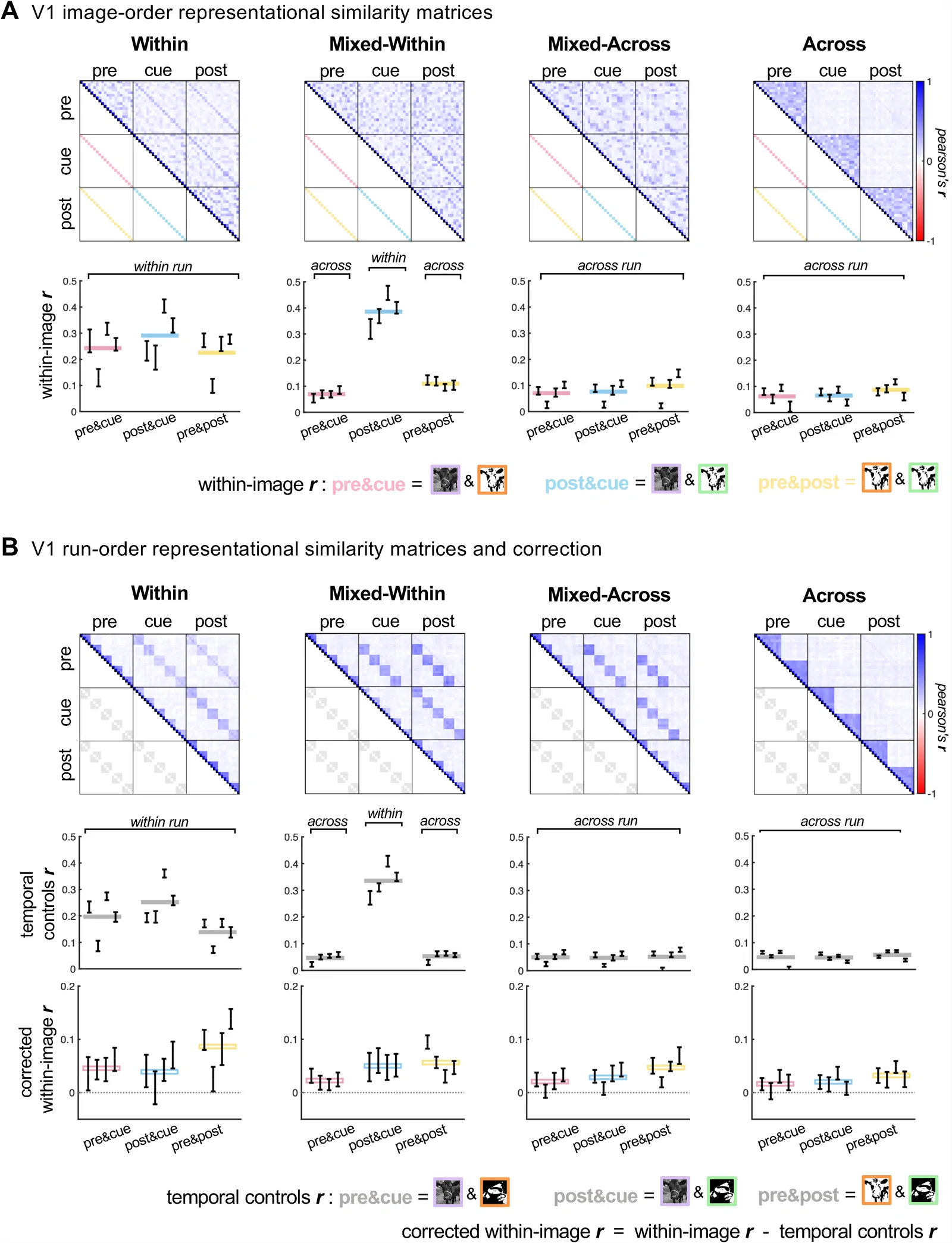
Temporal order effects correlate with RSA-derived measures of perceptual reorganisation in primary visual cortex. (A) Top row: Group-averaged image-order RSMs for primary visual cortex (V1), ordered by stimulus condition (Pre, Cue and Post) and image identity (Im_1_ to Im_20_) for each paradigm group. Correlations of the same image presented in different conditions are represented along RSM off-centre diagonals (pink diagonal for Pre&Cue, blue diagonal for Post&Cue, and yellow diagonal for Pre&Post), where greater representational similarity is expected due to shared image identities, denoted by darker blue colours, compared to for different images. Bottom row: Mean within-image similarities for across-condition comparisons in V1. Error bars show bootstrapped 95% confidence intervals for individual subjects, horizontal coloured bars show group averages for each across-condition comparison. (B) Top row: Group-averaged run-order RSMs for V1, ordered by stimulus condition (Pre, Cue, and Post) and experiment presentation order. Grey squares indicate temporal controls for each paradigm: pairs of non-corresponding images that appear in the same fMRI runs as the corresponding images in each across-condition comparison. Middle row: Mean similarities of temporal controls for across-condition comparisons in V1. Error bars show bootstrapped 95% confidence intervals for individual subjects, horizontal grey bars show group averages for each across-condition comparison. Bottom row: Correction of mean similarities for across-condition comparisons of corresponding images in V1. For each across-condition comparison, within-image similarities are corrected by subtracting mean similarities of temporal controls within each image set and ROI at the single-subject level. Error bars show bootstrapped 95% confidence intervals for individual subjects, horizontal hollow bars show group averages for each across-condition comparison.

Under perceptual reorganisation, the neural representation of a two-tone image after cueing (Post) is expected to more closely match the representation of its original photo (Cue). Hence, the correlation between cued two-tones and their corresponding photo cues (Post&Cue, Fig. 2A, blue diagonals) should be higher than for uncued two-tones and their corresponding photo cues (Pre&Cue, Fig. 2A, pink diagonals). Positive patterns of perceptual reorganisation are, therefore, defined as Post&Cue > Pre&Cue. Paired t-tests across images at the single-subject level reveal highly significant perceptual reorganisation effects in the Mixed-Within paradigm (Post&Cue vs Pre&Cue: t(19) > 12, Cohen’s *dz* > 2.6, *p* < 10^-9^), a more moderate effect in the Within paradigm (t(19) > 2.4, Cohen’s *dz* > 0.56, *p* < 0.05 for three of four subjects), and a nonsignificant effect in across-run designs (*p* > 0.05 for all subjects; see Supplementary Table S1 for all statistical results). These discrepancies in the presence of a neural perceptual reorganisation effect occurred despite all paradigms producing a large behavioural effect (see Supplementary Text S1).

Across all paradigms, we find a strong influence of the temporal distribution of stimulus presentations on RSA measures of visual representations. For example, across all paradigms, correlations of corresponding images across conditions that were presented within the same run (within-run) were higher than those presented in different runs (between-run; Fig. 2A; linear mixed model: β(within/between) = 0.324, SE = 0.009, t(954) = 34.319, *p* < e-100, 95% CI [0.31, 0.34]). This is especially problematic in the Mixed-Within paradigm, where Post&Cue comparisons of corresponding images are always within a run, while Pre&Cue comparisons are always across runs, as this confounds the key contrast for perceptual reorganisation with temporal order structure in the BOLD signal.

Temporal artefacts can arise at multiple timescales across an experiment. For example, in addition to the within- versus across-run differences we report above, correlations may also drift within a run or across the session. Therefore, all four paradigms could be affected by temporal consistencies in stimulus presentation sequences. To explore this, we next ran two analyses to assess for perceptual reorganisation effects in pairwise image comparisons with equivalent temporal relationships as in the above analysis, but that would not be expected to show visually driven effects. Firstly, in a control cortical ROI (primary somatosensory cortex [S1]), and secondly, in V1 representations of non-corresponding images. Because perceptual reorganisation reflects knowledge-based modulation of visual representations, we would not expect to find neural markers of this effect in primary somatosensory cortex (S1). However, in this non-visual ROI, we nevertheless found strong patterns of perceptual reorganisation in the Within and Mixed-Within paradigms (Fig. 3A). Mirroring findings from V1, Post&Cue correlations exceeded Pre&Cue correlations at the single-subject level in the Mixed-Within design (paired t-tests: t(19) > 11, Cohen’s *dz* *>* 2.3, *p* < 10^-8^) and the Within design (t(19) > 4.2, Cohen’s *dz* > 0.95, *p* < 0.001 for three of four subjects; see Supplementary Table S1 for all statistical results). The presence of equivalently large perceptual reorganisation effects from visual stimuli in the early somatosensory cortex indicates that this marker is not constrained to visual areas, but may be susceptible to methodological artefacts that falsely emulate neurocognitive learning effects beyond the visual cortex.

**Fig. 3.**
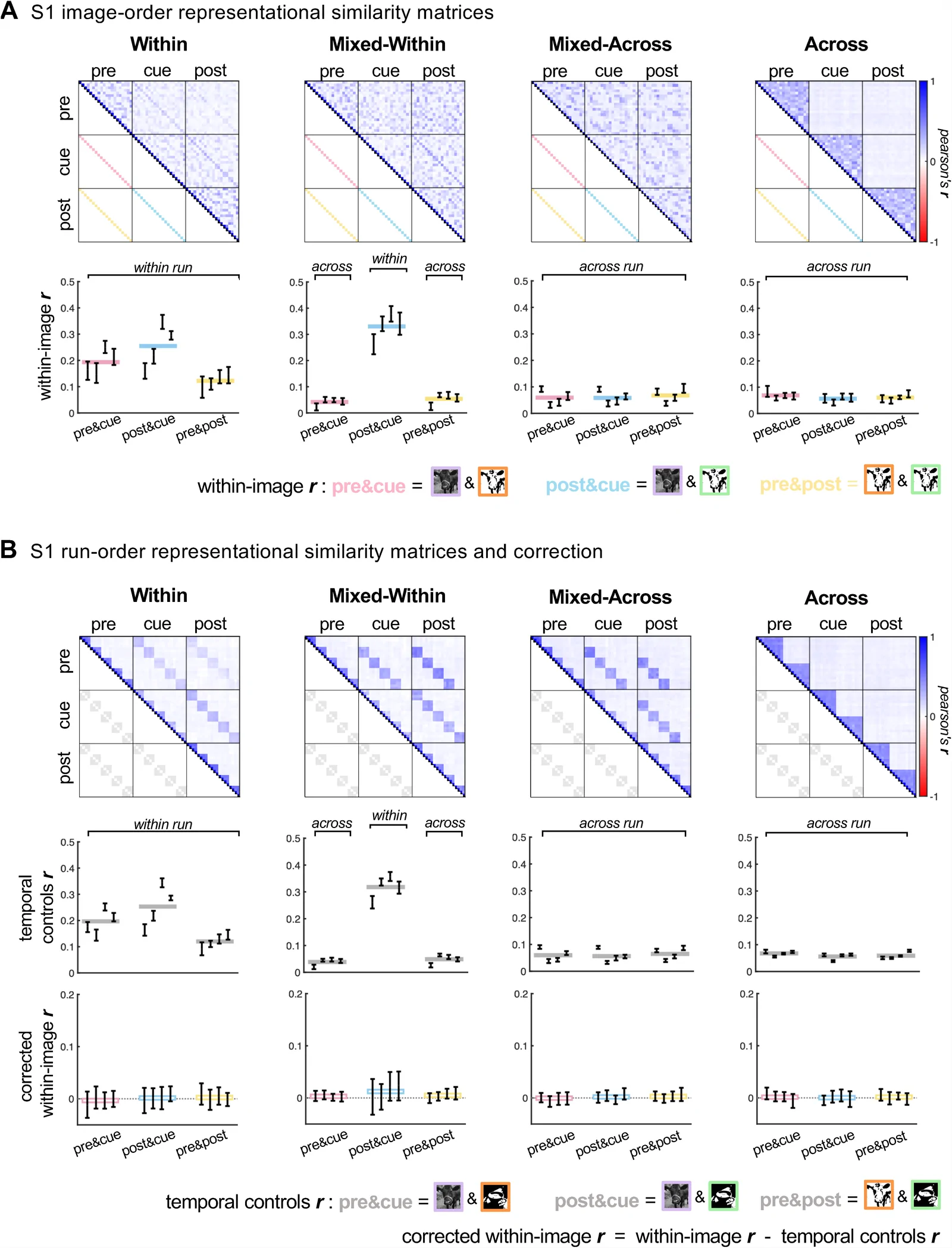
Temporal order effects extend beyond visual cortex, emulating perceptual reorganisation in the primary somatosensory cortex. (A) Top row: Group-averaged image-order RSMs for primary somatosensory cortex (S1), ordered by stimulus condition (Pre, Cue, and Post) and image identity (Im_1_ to Im_20_) for each paradigm group. Correlations of the same image presented in different conditions are represented along RSM off-centre diagonals (pink diagonal for Pre&Cue, blue diagonal for Post&Cue, and yellow diagonal for Pre&Post), where greater representational similarity is expected, denoted by darker blue colours, compared to for different images. Bottom row: Mean within-image similarities for across-condition comparisons in S1. Error bars show bootstrapped 95% confidence intervals for individual subjects, horizontal coloured bars show group averages for each across-condition comparison. (B) Top row: Group-averaged run-order RSMs for S1, ordered by stimulus condition (Pre, Cue and Post) and experiment presentation order. Grey squares indicate temporal controls for each paradigm: pairs of non-corresponding images that appear in the same fMRI runs as the corresponding images in each across-condition comparison. Middle row: Mean similarities of temporal controls for across-condition comparisons in S1. Error bars show bootstrapped 95% confidence intervals for individual subjects, horizontal grey bars show group averages for each across-condition comparison. Bottom row: Correction of mean similarities for across-condition comparisons of corresponding images in S1. For each across-condition comparison, within-image similarities are corrected by subtracting mean similarities of temporal controls within each image set and ROI at the single-subject level. Error bars show bootstrapped 95% confidence intervals for individual subjects, horizontal hollow bars show group averages for each across-condition comparison.

We next considered how temporal order structure in the BOLD signal may impact representational similarity measures of perceptual reorganisation across the different paradigms we tested. To explore this, we constructed additional ‘run-order RSMs’ (see Section 2; Fig. 2B) that are ordered according to when each image was presented across the experiment—which differed for every subject due to trial pseudo-randomisation. Rows and columns of RSMs are arranged by stimulus condition (Pre, Cue, and Post), and, conventionally, are arranged within each condition by a consistent pre-defined order of images across all subjects, regardless of the order in which the images were presented (Fig. 2A, image-order RSMs). In a run-order RSM, images are arranged by the unique order in which they were presented to each subject, such that the first rows include the images presented first, but the identity of these images will differ for each subject. Notably, run-order RSMs still display correlations of corresponding images along the off-centre diagonals, because image presentation order was consistent across conditions, but in addition, these matrices will visualise any temporally driven correlation structures that would otherwise be obscured by image-order RSMs (see Fig. 1C).

Group-averaged run-order RSMs for V1 are shown in Figure 2B. In addition to increased correlations between corresponding images across different conditions previously visible in image-order RSMs, the run-order RSMs also reveal temporal correlation structure unrelated to stimulus identity. Most notably, multi-voxel patterns from any two images presented in the same fMRI run are clearly more correlated than those presented in different runs across all paradigms. In run-order RSMs, this appears as dark blue blocks for any comparisons made within a run, forming patterns that consistently reflect the paradigm’s run structure in single-subject RSMs (see Supplementary Text S2). This is a well-known feature of fMRI BOLD timeseries measures (Mumford et al., 2014), and becomes more discriminable after correction for common noise across the session (see Supplementary Text S3), but is also clearly present in our data before noise normalisation (Supplementary Fig. S3). This contextualises the earlier reported finding of increased correlations for corresponding images within- versus across-run, with run-order RSMs revealing structured correlation increases that extend to non-corresponding images. These matrices are useful for visualising how the arrangement of conditions across fMRI runs can confound perceptual learning indices (here Post&Cue>Pre&Cue) in the presence of temporal correlations. For example, it becomes clear how a within- versus across-run correlation structure would artefactually inflate the perceptual reorganisation effect in the Mixed-Within design. The key comparison of Post&Cue versus Pre&Cue correlations of corresponding images becomes imbalanced, as Post and Cue conditions always fall within a run in this design, and so are artefactually enhanced, while comparisons of Pre and Cue conditions are always made across different runs.

To assess the impact of temporal artefacts on RSA measures of perceptual reorganisation, we used a control measure that isolates the effects of temporal experiment structure on multi-voxel pattern correlations from those relating to image identity. Specifically, for each comparison of corresponding images across conditions (i.e., Pre&Cue, Post&Cue, and Pre&Post), we calculated the average representational similarity of non-corresponding images with equivalent temporal relationships, and used these as temporal controls (light grey squares in RSMs in Figs. 2B and 3B). Because these temporal controls include images from the same conditions and runs as the main within-image comparisons, they should capture the same temporal correlation structure, but not the perceptual learning effects that are specific to the shared identity of corresponding images. This analysis revealed that in the Within and Mixed-Within designs, temporal-order artefacts produce a correlation structure that mimics a pattern of perceptual reorganisation; for temporal controls, Post&Cue similarity is significantly higher than Pre&Cue similarity in both V1 and S1 in these designs (Fig. 2B; Mixed-Within, paired t-tests: t(59) > 17, Cohen’s *dz* > 2, *p* < 10^-25^; Within: t(59) > 4, Cohen’s *dz* > 0.5, *p* < 10^-4^ for three of four subjects; see Supplementary Table S2 for all statistical results). These control analyses reveal temporal artefacts that exert significant and equivalent influences across visual and non-visual cortical ROIs.

Next, to isolate image-driven neural effects from temporally driven artefacts, we corrected comparisons of interest by subtracting the mean correlation of temporal controls on a run-by-run basis. This results in a similarity measure that appropriately corrects for the temporal confounds that each image comparison is subject to across multiple timescales, while isolating image-specific changes in neural representations (see Section 2). At the single-subject level, we found that this correction removed significant patterns of perceptual reorganisation in V1, which were previously present in seven individuals, leaving this pattern in just one out of these seven (Fig. 2B, bottom panel; see Supplementary Table S3 for all statistical results). While we cannot determine from these results whether perceptual reorganisation of visual representations is present in V1, as effects may be too small to detect at the single-subject level, we can confirm that the large effect sizes we previously observed using conventional RSA measures were erroneous.

In S1, correcting for temporal artefacts resulted in near-zero correlations across all conditions and paradigms, abolishing any previous evidence of visual perceptual reorganisation in this non-visual brain area at the single-subject level (Fig. 3B, bottom panel; *p* > 0.05 for all comparisons). This shows that multivariate fMRI measures of perceptual learning could be strongly inflated by non-specific temporal order effects when not accounted for with appropriate controls.

## 4 Discussion

Perceptual learning, where new information alters neural representations, is widely studied with multivariate fMRI. Here, we demonstrate how temporal order effects can mimic such learning in the brain, by biasing conventional measures using representational similarity analysis (RSA; Kriegeskorte et al., 2008), revealing a critical vulnerability of some perceptual learning paradigms. Temporal artefacts, if not properly addressed, can masquerade as genuine perceptual effects, yet appropriate controls are rarely reported. This raises the possibility that some findings attributed to perceptual learning may be inflated or artefactual. To address this, we propose three methodological safeguards for multivariate fMRI studies of learning: (1) minimising order confounds, (2) controlling for temporal artefacts, and (3) checking that measured effects are specific to cognitive processes.

We tested four fMRI cueing paradigms, including examples widely used in the literature, designed to elicit perceptual reorganisation—the recognition of a previously ambiguous two-tone image after cueing with the original photo version. All paradigms featured the same basic structure: naïvely viewed two-tone images (‘Pre’), followed by their corresponding photo cues (‘Cue’), and the same two-tones viewed again after cueing (‘Post’). Our key manipulation was how these conditions were distributed across fMRI runs, which strongly shaped the results.

Several previous studies have reported striking perceptual reorganisation of two-tone image representations by prior knowledge (Flounders et al., 2019; González-García et al., 2018; González-García & He, 2021; Gorlin et al., 2012; Hsieh et al., 2010; Lazarova et al., 2025; van Loon et al., 2016), but do not consistently account for temporal order, and other non-specific, effects. Using conventional RSA approaches, we found large and significant perceptual reorganisation effects in V1 at the single-subject level for some designs that were small or absent in others, despite all paradigms inducing a strong behavioural effect. Strikingly similar patterns appeared in a control region, the primary somatosensory cortex (S1), which is not usually involved in visual scene parsing. A genuine neural signature of perceptual reorganisation should (a) replicate across fMRI paradigms that elicit strong behavioural effects—notwithstanding potential differences in design efficiency, and (b) manifest most strongly in cortical areas associated with the relevant sensory domain. As neither criterion was met in our data, we suspected a methodological rather than cognitive driver of the perceptual reorganisation results in V1.

It is well known that fMRI is subject to multiple artefacts in the temporal domain (Aguirre et al., 1997; Bianciardi et al., 2009; Mumford et al., 2014; Power et al., 2012). Recent studies have reiterated the need to apply controls that fully account for temporal artefacts, such as trial randomisation (Alink et al., 2015; Cai et al., 2019; Mumford et al., 2014), or corrective analyses (Cai et al., 2019; Lee & Rideaux, 2025). However, as perceptual learning paradigms require fixed condition orders, only partial randomisation is possible. Multiple temporal order confounds are likely to affect a single dataset simultaneously (e.g., drift in multi-voxel pattern correlations within- versus across-runs, over time within a run, and across the entire session), and their severity may vary from study to study depending on the scanner, sequence, design, and data processing steps. As such, choosing a specific design (e.g., the across-run design) that minimises a particular temporal correlation structure (e.g., within-run correlation structure) may not always be appropriate, especially when accounting for additional experimental constraints. Perceptual learning paradigms, for example, may depend on cue and target stimuli being presented in close temporal proximity to trigger a learning effect. We, therefore, propose a targeted, flexible framework consisting of three key steps.

Firstly, we recommend visualising the temporal correlation structure of fMRI RSA data for any study permitting only partial trial randomisation to ensure that the contrasts of interest are minimally confounded with potential temporal artefacts in the paradigm design. As we show in Figure 1C, partial randomisation—for example, randomising image order but not condition—can obscure temporal confounds without removing them. This is because a conventional ordering of RSMs (i.e., by image identity) randomly scatters temporally ordered correlation structure across the RSM. Critically, because non-corresponding image pairs may have different temporal placements within the session for different participants (i.e., two images presented in the same run for subject A may appear in different runs for subject B), any confounds driven by when non-corresponding images are shown may be reduced when averaging image-ordered RSMs across subjects. In contrast, matching images always appear in a consistent temporal relationship, so temporal order biases for these images will remain, even at the group level. In our data, visualising RSMs by presentation order in small pilot samples revealed strong temporal correlation structures at the individual participant level that were present in all paradigms but hidden in conventional analyses. Crucially, this made clear how design choices interacted with these patterns. In our data, when placing key condition comparisons within the same fMRI run, the inherent correlation structure of the BOLD measure, which was consistently more correlated for within- than across-run image pairs, produced a clear artefactual pattern resembling perceptual reorganisation (Figs. 2B and 3B). Thus, constructing run-order RSMs for a small number of pilot scans or simulated data can offer critical information for design optimisation.

As a second step, we suggest applying controls for non-specific effects such as temporal order confounds. Even after avoiding the most pronounced temporal confounds at the design stage, more subtle temporal correlation structures may still bias RSA results. It is therefore useful to model these during the analysis stage in order to isolate genuine learning-related changes. For example, potential temporal correlation structures (e.g., trial number, run number, or trial-by-trial separation) can be included as nuisance predictors to be regressed out of RSMs via linear modelling (Alink et al., 2015). In the current study, we calculated temporally matched controls that shared the same temporal correlation structures as the main comparisons (across-condition image pairs), but did not include corresponding image pairs, and so lacked image-specific perceptual learning effects. These controls demonstrate the contribution of temporal effects acting on main comparisons, and provide a benchmark that must be exceeded if representational similarity changes reflect genuine perceptual reorganisation effects that are specific to the learnt image. Subtracting the mean correlations of temporal controls from the main comparisons removed artefactual effects that mimicked perceptual reorganisation in both primary visual cortex (V1) and a control region (S1), demonstrating that uncorrected RSA measures can be misleading, in our case particularly in within-run designs. Notably, after applying this correction, V1 multi-voxel patterns became significantly more correlated for repeated presentations of the same two-tone stimulus compared with presentations of two-tone and photo stimuli. This was not the case for S1, however, where all measures of image-specific effects fell to zero. These findings converge with the established function of V1 in encoding low-level visual features, and the general insensitivity of S1 to visual information.

Finally, whilst effective, temporal corrections alone may not fully eradicate effects of non-interest from fMRI analyses. This is because multiple cognitive and non-cognitive processes contribute to the fMRI signal at any one time, and these may interact in non-additive ways. We, therefore, recommend incorporating complementary measurements as negative controls to ensure that the effects measured are perceptually relevant. This could consist of a control ROI outside the pathways likely to be affected by the cognitive effect in question, as we apply here with S1. Additionally, behavioural measures of perception collected during scanning can be used to separate out trials where perceptual reorganisation effects should be weak or absent. For example, two-tones for which recognition was unaltered by the photo cue (either the cue was not needed to recognise the two-tone, or the cue was ineffective) should result in minimal learning effects in the brain. Notably, the control analysis we apply in the current study jointly functions as a negative control; comparing multi-voxel pattern correlations for corresponding images to those of non-corresponding images accounts not only for temporal order effects, but also for non-specific cognitive influences, such as attention or response suppression, that may apply differentially across stimulus conditions.

These recommendations not only help avoid temporal-order-related confounds, but also ensure that observed changes truly reflect the learning process in question. Here, we focused on how learning affects responses to individual images. In contrast, other two-tone studies have looked at broader effects that are independent of image identity. For example, two-tone images are reportedly encoded more distinctly to each other following photo cueing (González-García et al., 2018; Lazarova et al., 2025; van Loon et al., 2016), or that a cued two-tone becomes more similar to its corresponding photo cue without determining whether its similarity to a non-corresponding photo equally increased (Hsieh et al., 2010). These effects can be influenced by unrelated factors like attentional drift, repetition suppression, or adaptation (Alink et al., 2015; Cai et al., 2019; Grill-Spector et al., 2006), which over time may reduce within-condition correlations or increase apparent cue similarity without reflecting true learning. In contrast, image-specific contrasts and converging measures across neural and behavioural data better isolate content-driven perceptual changes, especially when combined with appropriate controls for global trends.

A limitation of our study is the small sample size of participants scanned with individual perceptual learning designs, which restricts our ability to differentiate confounds across paradigms. However, the point we aim to make is principled and pragmatic, not specific to the tested task or paradigms. Our data clearly reveal how temporal correlation structure confounds present in all the paradigms we test can be particularly harmful for certain designs. We demonstrate how even small N designs that are feasible to run as pilots can capture these inherent confounds, and be used to develop protocols that minimise and control for their unwarranted impact on conclusions.

After applying the above-recommended corrective steps, we no longer observed measurable perceptual reorganisation in V1 at the single-subject level. Instead, V1 representations of cued two-tone images were most similar to initial presentations of the same two-tones, consistent with low-level visual encoding of image features. Whether perceptual reorganisation of these stimuli is present at the level of V1 remains an open question, which this manuscript does not attempt to answer. While large reorganisation effects have been previously reported in the early visual cortex (Flounders et al., 2019; González-García et al., 2018; González-García & He, 2021; Hsieh et al., 2010; van Loon et al., 2016), it is not possible to determine whether these results are affected by the confounds presented here without additional control analyses. In our data, evidence of perceptual reorganisation in V1 and S1 was fully eliminated after applying appropriate corrections. We, therefore, recommend that all studies on neural mechanisms of learning—and perceptual reorganisation in particular—follow steps to minimise, correct, and check for non-specific effects, both temporal and cognitive, that can confound fMRI measures of learning over time. Accounting for temporal confounds in this way will enable accurate measures of genuine cognitive effects, and improve comparability and replicability across studies.

## Supplementary Material

Supplementary Material

## Data Availability

Analysis code, and behavioural and RSA data are available at www.github.com/ChildVisionLab/Temporal_Confounds
