# Supplementary material for: Temporal confounds emulate multivariate fMRI measures of perceptual learning
Source: Imaging Neurosci (Camb). 2026 Nov 4;4:IMAG.a.1435. doi: 10.1162/IMAG.a.1435 (PMC13736823; doi:10.1162/IMAG.a.1435)
Supplement: Supplementary file 1 [file IMAG.a.1435_supp.pdf]

Supplementary information

Supplementary Text S1: Behavioural perceptual reorganisation effect

All participants completed two behavioural measures during the experiment: verbal naming of two-tone content (completed between fMRI runs; Supplementary Figure S1, left plot), and image categorisation of two-tone images (animate/inanimate categorisation via button-press completed within each trial; Supplementary Figure S1, right plot). For each paradigm, generalised linear mixed models with random intercepts for subjects and images revealed a significant effect of two-tone condition on image recognition (β > 1.55, SE < 0.8, t > 4.07, $p < 10^{-4}$), and linear mixed models with the same random intercepts revealed a significant effect of two-tone condition on image categorisation (β > 0.08, SE < 0.04, t > 2.7, $p < 0.01$). These results reveal a large effect of photo cueing on two-tone recognition.

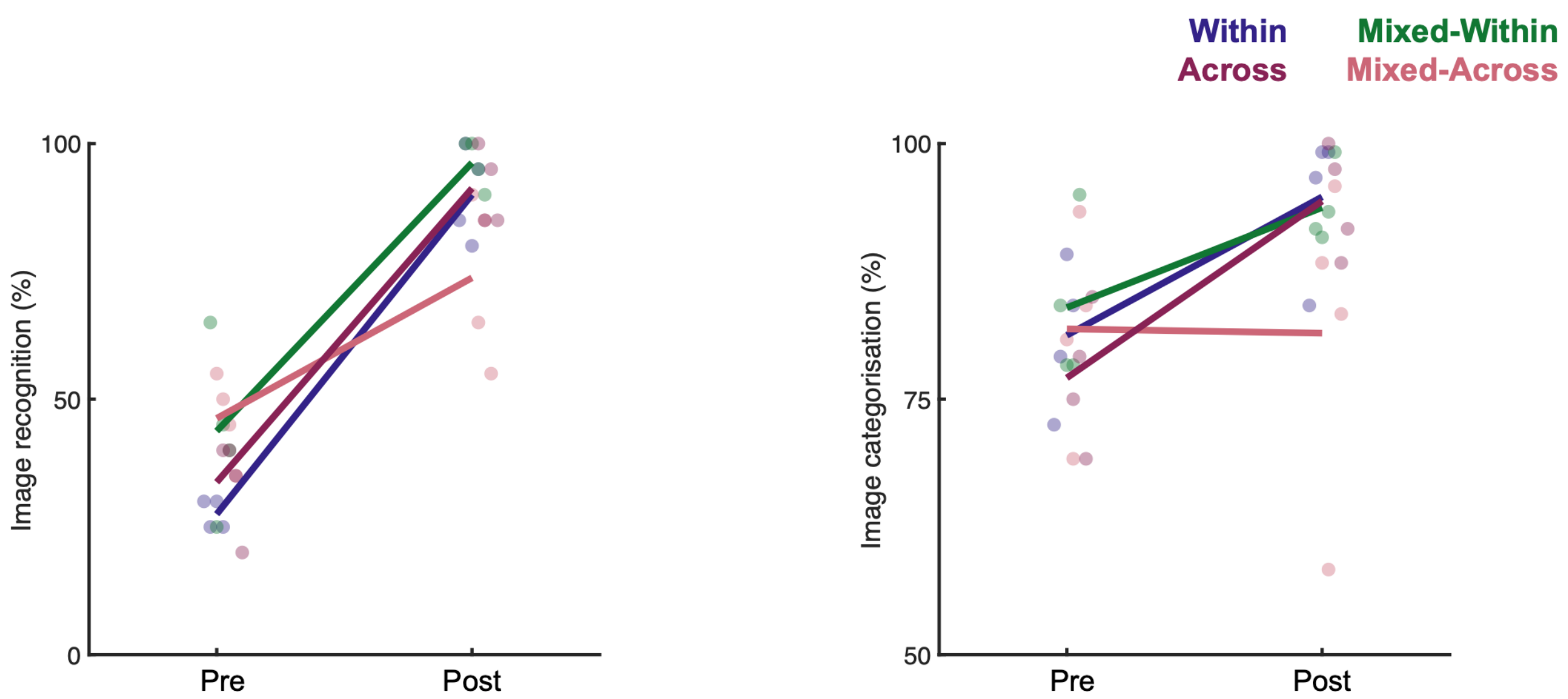

Supplementary Figure S1: Behavioural measures of perceptual reorganisation

Image recognition measured via between-run verbal naming of image content (left panel), and image categorisation, measured via within-trial animate/inanimate button-presses (right panel). Lines show mean cueing effect per paradigm (mean Post recognition – mean Pre recognition), and markers show individual subject performance.

Supplementary Text S2: Image- and run-order single-subject RSMs

Temporal order artefacts are substantial at the single-subject level, causing large amplifications of within-image similarities for any comparison made within a run (Supplementary Figure S2). These paradigm-dependent patterns are highly consistent at the single-subject level; within each paradigm, pairwise Pearson correlations between subjects' run-order RSM's were very high: V1 = 0.92, S1 = 0.94 (averaged across all paradigms), and significantly larger than image-order RSMs: V1 = 0.58, S1 = 0.57 (paired t-test between run-order and image-order pairwise correlations across all paradigms, for each ROI: $t(23) > 10$, Cohen's $dz > 2$, $p < 10^{-10}$).

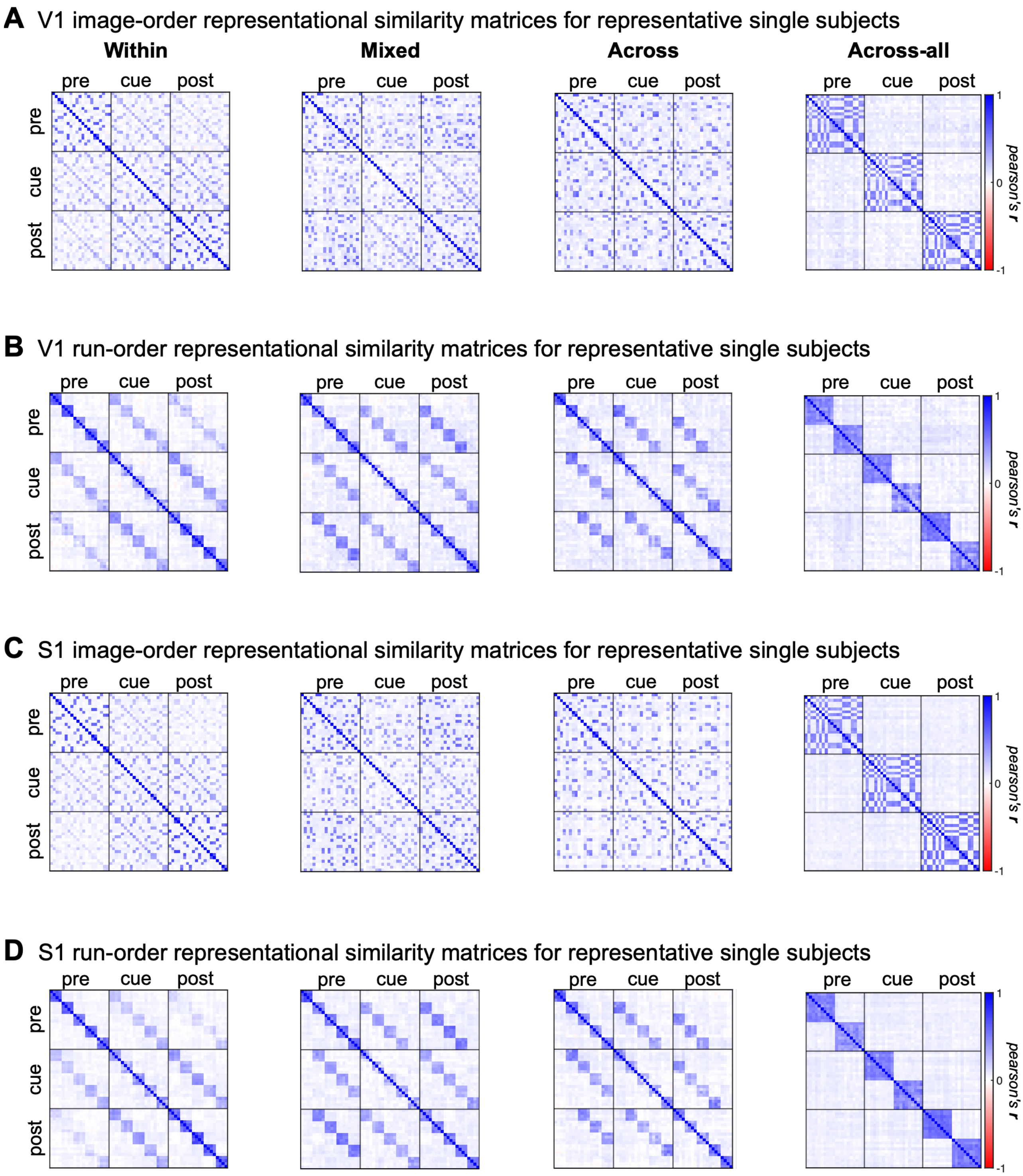

Supplementary Figure S2: Representative single-subject representational similarity matrices

A Representative single-subject image-order RSMs from each paradigm group shown for primary visual cortex (V1), ordered by stimulus condition (Pre, Cue and Post) and image identity (Im_1 to Im_{20}). **B** Representative single-subject run-order RSMs from each paradigm group for V1, ordered

by stimulus condition (Pre, Cue and Post) and experiment presentation order. Subjects are the same as in panel A. **C** Representative single-subject image-order RSMs from each paradigm group shown for primary somatosensory cortex (S1). Subjects are the same as in panel A. **D** Representative single-subject run-order RSMs from each paradigm group for S1. Subjects are the same as in panel A.

Supplementary Text S3: Unnormalised RSA measures of perceptual reorganisation

We applied a widely used multivariate normalisation of fMRI pattern data (Walther et al., 2016), which increases the signal-to-noise ratio (SNR) of our data by removing correlated noise from the data. After this normalisation, the temporal artefacts became more visually discernible in run-order RSMs. It is important to note, however, that these artefacts were not *introduced* by the multivariate normalisation step – below we present evidence that they become more detectible after normalisation because this procedure (a) increases SNR, and therefore power to detect the impacts of temporal order effects that (b) were already present in unnormalised data.

Firstly, as expected with lower SNR data, across-condition correlations of image pairs in the unnormalised analysis of V1 were on average higher and more variable than in the noise-normalised analysis across all subjects (increased mean and standard deviation in unnormalised compared to normalised: $t(15) > 4$, Cohen's $dz > 1$, $p < 0.001$). Further evidence for lower SNR in the unnormalised data can be seen when comparing corresponding image correlations (e.g., Pre image1 with Post image1) to correlations across noncorresponding images (e.g., Pre image1 and Post image2, also referred to as temporal controls). Across all paradigms, corresponding image correlations significantly exceeded temporal controls in only 5 of the 16 participants before applying noise normalisation; after normalisation, these differences became significant in 13 out of 16 participants ($p < 0.05$). Thus, multivariate normalisation increases SNR, rendering measures more sensitive to signals driven by stimulus manipulations as well as temporal correlation structure that varies across the timeseries.

Secondly, temporal order artefacts are already present in our unnormalised data (Supplementary Figure S3). Comparing across unnormalised data from all paradigms,

correlations of corresponding images across conditions presented within the same run (within-run) were higher than those presented in different runs (between-run; linear mixed model: β(within/between) = 0.17, SE = 0.038, t(954) = 4.45, $p < 10^{-5}$, 95% CI [0.094, 0.243]). The unnormalised data analysis showed similar artefactually enhanced effects of perceptual reorganisation (Post&Cue > Pre&Cue) in the across-condition comparisons of corresponding images, as well as in the temporal controls (Supplementary Figure S3A and S3B, 2nd rows). However, while these are numerically similar, they did not always reach significance at the single-subject level, likely due to lower SNR in the unnormalised data as described above. In the Mixed-Within paradigm, significant effects were present for all single-subject comparisons of temporal controls in V1, and 3 of 4 subjects in S1 ($p < 0.05$). In the Within paradigm, Post&Cue > Pre&Cue was significant in V1 temporal controls for two of the three subjects that showed a significant effect in the normalised data ($p < 0.05$) and trending for one ($p = 0.08$). In the Mixed-Across paradigm, no significant effects of perceptual reorganisation were present for any subjects in V1 temporal controls, but were present in one of four subjects in S1 temporal controls ($p < 0.05$). Meanwhile, for the Mixed-Across and Across paradigms, these effects reached significance in at least one ROI for three of eight subjects ($p < 0.05$). Together, this suggests the temporal artefacts observed in normalised data are also present at the single-subject level before applying normalisation.

After correcting for temporal order artefacts (see Methods), no significant perceptual reorganisation effects were found at the single-subject level for any paradigm in either ROI ($p > 0.05$ for all subjects).

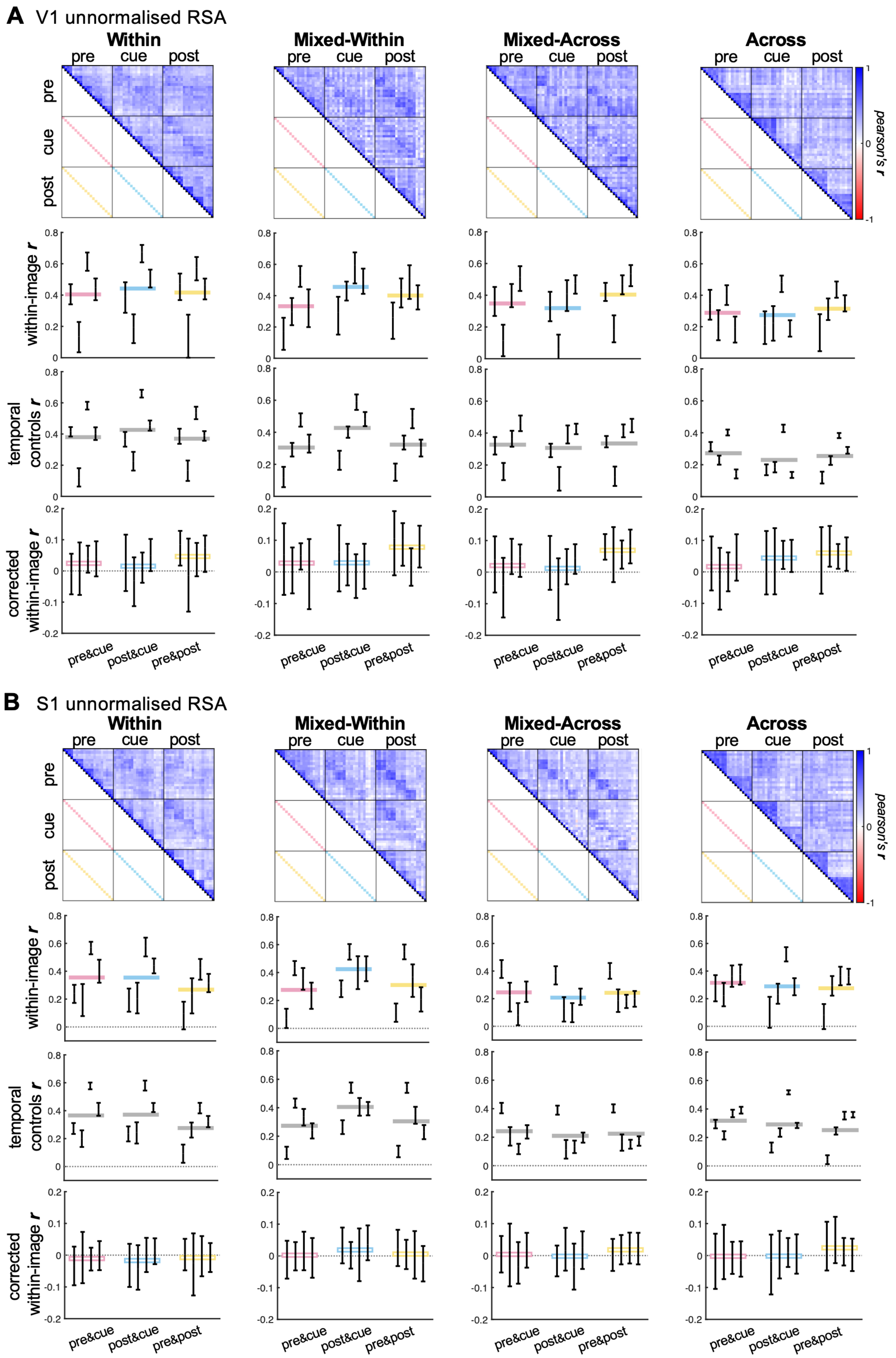

A V1 unnormalised RSA
B S1 unnormalised RSA
Within
Mixed-Within
Mixed-Across
Across
pre
cue
post
pearson's r
within-image r
temporal controls r
corrected within-image r
pre&cue
post&cue
pre&post

Supplementary Figure S3: Unnormalised representational similarity matrices and measures of perceptual reorganisation

A First row: Group-averaged run-order RSMs of unnormalised multi-voxel response patterns for primary visual cortex (V1), ordered by stimulus condition (Pre, Cue and Post) and experiment presentation order. 2nd row: Mean within-image similarities for across-condition comparisons of unnormalised multi-voxel response patterns in V1. Error bars show bootstrapped 95% confidence intervals for individual subjects, horizontal coloured bars show group averages for each across-condition comparison. 3rd row: Mean similarities of temporal controls for across-condition comparisons of unnormalised multi-voxel response patterns in V1. Error bars show bootstrapped 95% confidence intervals for individual subjects, horizontal grey bars show group averages for each across-condition comparison. 4th row: Correction of mean similarities for across-condition comparisons of unnormalised multi-voxel response patterns to corresponding images in V1. For each across-condition comparison, within-image similarities are corrected by subtracting mean similarities of temporal controls within each image set and ROI at the single-subject level. Error bars show bootstrapped 95% confidence intervals for individual subjects, horizontal hollow bars show group averages for each across-condition comparison. **B** As in panel A, but for unnormalised multi-voxel response patterns for primary somatosensory cortex (S1).

Supplementary Table S1: Single-subject statistical tests for mean representational similarity of within-image comparison

	V1	Pre&Cue vs. Post&Cue	Post&Cue vs. Pre&Post	Pre&Cue vs. Pre&Post
Within	subject 1	**p = 0.019, q = 0.036, t(19) = 2.6, Cohen's dz = 0.57, SD: 0.071**	**p = 0.0091, q = 0.019, t(19) = -2.9, Cohen's dz = -0.65, SD: 0.066**	p = 0.91, q = 0.94, t(19) = -0.11, Cohen's dz = -0.025, SD: 0.089
Within	subject 2	**p = 0.02, q = 0.038, t(19) = -2.5, Cohen's dz = -0.57, SD: 0.14**	**p = 0.0017, q = 0.0044, t(19) = 3.6, Cohen's dz = 0.82, SD: 0.13**	p = 0.076, q = 0.12, t(19) = 1.9, Cohen's dz = 0.42, SD: 0.07
Within	subject 3	**p = 1.9e-06, q = 8.1e-06, t(19) = -6.7, Cohen's dz = -1.5, SD: 0.057**	**p = 1.8e-09, q = 1.1e-08, t(19) = 11, Cohen's dz = 2.4, SD: 0.061**	**p = 1e-04, q = 3.9e-04, t(19) = 4.9, Cohen's dz = 1.1, SD: 0.055**
Within	subject 4	**p = 0.002, q = 0.0048, t(19) = -3.6, Cohen's dz = -0.8, SD: 0.085**	**p = 0.016, q = 0.031, t(19) = 2.6, Cohen's dz = 0.59, SD: 0.087**	p = 0.22, q = 0.31, t(19) = -1.3, Cohen's dz = -0.28, SD: 0.058
Mixed-Within	subject 5	**p = 2.9e-10, q = 2e-09, t(19) = -12, Cohen's dz = -2.7, SD: 0.1**	**p = 5.9e-09, q = 3.1e-08, t(19) = 9.9, Cohen's dz = 2.2, SD: 0.089**	**p = 4.9e-08, q = 2.4e-07, t(19) = -8.7, Cohen's dz = -1.9, SD: 0.035**
Mixed-Within	subject 6	**p = 3.5e-17, q = 3e-15, t(19) = -29, Cohen's dz = -6.5, SD: 0.046**	**p = 6.1e-14, q = 6.5e-13, t(19) = 19, Cohen's dz = 4.3, SD: 0.058**	**p = 1.3e-05, q = 5.2e-05, t(19) = -5.8, Cohen's dz = -1.3, SD: 0.039**
Mixed-Within	subject 7	**p = 2e-16, q = e-14, t(19) = -26, Cohen's dz = -5.9, SD: 0.066**	**p = 2e-15, q = 4.6e-14, t(19) = 23, Cohen's dz = 5.2, SD: 0.07**	**p = 0.0061, q = 0.013, t(19) = -3.1, Cohen's dz = -0.69, SD: 0.034**
Mixed-Within	subject 8	**p = 6e-15, q = 9.6e-14, t(19) = -22, Cohen's dz = -4.9, SD: 0.064**	**p = 1.6e-14, q = 1.9e-13, t(19) = 21, Cohen's dz = 4.7, SD: 0.063**	p = 0.054, q = 0.09, t(19) = -2.1, Cohen's dz = -0.46, SD: 0.041
Mixed-Across	subject 9	p = 0.23, q = 0.32, t(19) = -1.3, Cohen's dz = -0.28, SD: 0.038	**p = 0.024, q = 0.044, t(19) = -2.5, Cohen's dz = -0.55, SD: 0.043**	**p = 1.3e-04, q = 4.5e-04, t(19) = -4.8, Cohen's dz = -1.1, SD: 0.032**
Mixed-Across	subject 10	p = 0.94, q = 0.95, t(19) = -0.077, Cohen's dz = -0.017, SD: 0.035	p = 0.65, q = 0.77, t(19) = 0.46, Cohen's dz = 0.1, SD: 0.041	p = 0.67, q = 0.79, t(19) = 0.43, Cohen's dz = 0.095, SD: 0.038
Mixed-Across	subject 11	p = 0.51, q = 0.63, t(19) = -0.67, Cohen's dz = -0.15, SD: 0.05	**p = 0.0043, q = 0.0099, t(19) = -3.2, Cohen's dz = -0.72, SD: 0.038**	**p = 0.0019, q = 0.0048, t(19) = -3.6, Cohen's dz = -0.8, SD: 0.043**
Mixed-Across	subject 12	p = 0.57, q = 0.68, t(19) = -0.58, Cohen's dz = -0.13, SD: 0.036	**p = 4.4e-04, q = 0.0012, t(19) = -4.2, Cohen's dz = -0.95, SD: 0.043**	**p = 2.9e-04, q = 8.8e-04, t(19) = -4.4, Cohen's dz = -0.99, SD: 0.046**
Across	subject 13	p = 0.98, q = 0.98, t(19) = -0.026, Cohen's dz = -0.0057, SD: 0.041	p = 0.78, q = 0.86, t(19) = 0.29, Cohen's dz = 0.064, SD: 0.036	p = 0.85, q = 0.9, t(19) = 0.19, Cohen's dz = 0.043, SD: 0.048
Across	subject 14	p = 0.82, q = 0.88, t(19) = -0.23, Cohen's dz = -0.051, SD: 0.043	**p = 2.6e-04, q = 8e-04, t(19) = -4.5, Cohen's dz = -1, SD: 0.038**	**p = 0.0011, q = 0.0029, t(19) = -3.8, Cohen's dz = -0.86, SD: 0.047**
Across	subject 15	p = 0.48, q = 0.6, t(19) = 0.71, Cohen's dz = 0.16, SD: 0.042	**p = 2.2e-04, q = 7.2e-04, t(19) = -4.5, Cohen's dz = -1, SD: 0.029**	**p = 0.0094, q = 0.019, t(19) = -2.9, Cohen's dz = -0.65, SD: 0.035**
Across	subject 16	p = 0.11, q = 0.18, t(19) = -1.7, Cohen's dz = -0.37, SD: 0.045	**p = 0.068, q = 0.11, t(19) = -1.9, Cohen's dz = 0.43, SD: 0.051**	**p = 0.015, q = 0.029, t(19) = -2.7, Cohen's dz = -0.6, SD: 0.065**

	S1	Pre&Cue vs. Post&Cue	Post&Cue vs. Pre&Post	Pre&Cue vs. Pre&Pos
Within	subject 1	p = 0.78, q = 0.86, t(19) = 0.28, Cohen's dz = 0.064, SD: 0.089	**p = 0.0087, q = 0.019, t(19) = 2.9, Cohen's dz = 0.65, SD: 0.09**	p = 0.035, q = 0.061, t(19) = 2.3, Cohen's dz = 0.51, SD: 0.13
Within	subject 2	**p = 2.3e-04, q = 7.2e-04, t(19) = -4.5, Cohen's dz = -1, SD: 0.073**	**p = 9.5e-09, q = 4.8e-08, t(19) = 9.6, Cohen's dz = 2.2, SD: 0.052**	p = 0.033, q = 0.058, t(19) = 2.3, Cohen's dz = 0.52, SD: 0.073
Within	subject 3	**p = 3.9e-04, q = 0.0011, t(19) = -4.3, Cohen's dz = -0.96, SD: 0.1**	**p = 2.5e-10, q = 1.8e-09, t(19) = 12, Cohen's dz = 2.7, SD: 0.078**	**p = 4e-07, p = 1,8e-06, t(19) = 7.5, Cohen's dz = 1.7, SD: 0.068**
Within	subject 4	**p = 4.2e-04, q = 0.0012, t(19) = -4.3, Cohen's dz = -0.95, SD: 0.085**	**p = 2.1e-07, q = 9.5e-07, t(19) = 7.9, Cohen's dz = 1.8, SD: 0.085**	**p = 1.9e-05, p = 7.1e-05, t(19) = 5.7, Cohen's dz = 1.3, SD: 0.054**
Mixed-Within	subject 5	**p = 2e-09, q = 1.1e-08, t(19) = -11, Cohen's dz = -2.4, SD: 0.1**	**p = 2e-09, q = 1.1e-08, t(19) = 11, Cohen's dz = 2.4, SD: 0.1**	p = 0.75, q = 0.85, t(19) = -0.32, Cohen's dz = -0.072, SD: 0.038
Mixed-Within	subject 6	**p = 7.4e-15, q = e-13, t(19) = -22, Cohen's dz = -4.8, SD: 0.06**	**p = 3.4e-13, q = 3.3e-12, t(19) = 18, Cohen's dz = 3.9, SD: 0.069**	**p = 0.012, q = 0.025, t(19) = -2.8, Cohen's dz = -0.62, SD: 0.028**
Mixed-Within	subject 7	**p = e-15, q = 4.6e-14, t(19) = -24, Cohen's dz = -5.3, SD: 0.062**	**p = 5e-15, q = 8.8e-14, t(19) = 22, Cohen's dz = 5, SD: 0.063**	**p = 0.0047, q = 0.011, t(19) = -3.2, Cohen's dz = -0.72, SD: 0.023**
Mixed-Within	subject 8	**p = 5.9e-11, q = 5.1e-10, t(19) = -13, Cohen's dz = -2.9, SD: 0.1**	**p = 1.1e-10, q = 9.1e-10, t(19) = 13, Cohen's dz = 2.8, SD: 0.1**	p = 0.17, q = 0.26, t(19) = -1.4, Cohen's dz = -0.32, SD: 0.036
Mixed-Across	subject 9	p = 0.79, q = 0.86, t(19) = 0.27, Cohen's dz = 0.061, SD: 0.027	p = 0.24, q = 0.33, t(19) = 1.2, Cohen's dz = 0.27, SD: 0.037	p = 0.19, q = 0.27, t(19) = 1.4, Cohen's dz = 0.31, SD: 0.038
Mixed-Across	subject 10	p = 0.37, q = 0.47, t(19) = -0.92, Cohen's dz = -0.21, SD: 0.03	p = 0.8, q = 0.86, t(19) = -0.26, Cohen's dz = -0.058, SD: 0.03	p = 0.32, q = 0.43, t(19) = -1, Cohen's dz = -0.23, SD: 0.035
Mixed-Across	subject 11	p = 0.93, q = 0.95, t(19) = -0.087, Cohen's dz = -0.019, SD: 0.044	p = 0.16, q = 0.25, t(19) = -1.4, Cohen's dz = -0.32, SD: 0.043	p = 0.13, q = 0.21, t(19) = -1.6, Cohen's dz = -0.35, SD: 0.042
Mixed-Across	subject 12	p = 0.32, q = 0.43, t(19) = 1, Cohen's dz = 0.23, SD: 0.041	**p = 0.0027, q = 0.0063, t(19) = -3.5, Cohen's dz = -0.77, SD: 0.041**	p = 0.041, q = 0.07, t(19) = -2.2, Cohen's dz = -0.49, SD: 0.045
Across	subject 13	**p = 1.2e-04, q = 4.4e-04, t(19) = 4.8, Cohen's dz = 1.1, SD: 0.023**	p = 0.89, q = 0.92, t(19) = 0.15, Cohen's dz = 0.033, SD: 0.031	**p = 0.002, q = 0.0048, t(19) = 3.6, Cohen's dz = 0.8, SD: 0.032**
Across	subject 14	p = 0.036, q = 0.061, t(19) = 2.3, Cohen's dz = 0.51, SD: 0.034	p = 0.29, q = 0.4, t(19) = -1.1, Cohen's dz = -0.24, SD: 0.036	p = 0.37, q = 0.47, t(19) = 0.92, Cohen's dz = 0.21, SD: 0.042
Across	subject 15	p = 0.56, q = 0.68, t(19) = 0.6, Cohen's dz = 0.13, SD: 0.032	p = 0.68, q = 0.79, t(19) = 0.41, Cohen's dz = 0.093, SD: 0.033	p = 0.23, q = 0.32, t(19) = 1.2, Cohen's dz = 0.28, SD: 0.027
Across	subject 16	p = 0.73, q = 0.83, t(19) = 0.35, Cohen's dz = 0.079, SD: 0.034	p = 0.14, q = 0.21, t(19) = -1.5, Cohen's dz = -0.34, SD: 0.036	p = 0.36, q = 0.46, t(19) = -0.95, Cohen's dz = -0.21, SD: 0.041

Supplementary Table S2: Single-subject statistical tests for mean representational similarity of temporal controls

V1		Pre&Cue vs. Post&Cue	Post&Cue vs. Pre&Post	Pre&Cue vs. Pre&Post
Within	subject 1	**p = 1.9e-04, q = 3.4e-04, t(59) = 4, Cohen's dz = 0.45, SD: 0.085**	**p = 0.034, q = 0.048, t(59) = 2.2, Cohen's dz = 0.24, SD: 0.069**	**p = 1e-07, q = 2.6e-07, t(59) = 6.1, Cohen's dz = 0.68, SD: 0.08**
Within	subject 2	**p = 7.7e-09, q = 2.1e-08, t(59) = -6.7, Cohen's dz = -0.75, SD: 0.13**	**p = 1.1e-12, q = 3.9e-12, t(59) = 9, Cohen's dz = 1, SD: 0.11**	p = 0.24, q = 0.29, t(59) = 1.2, Cohen's dz = 0.13, SD: 0.085
Within	subject 3	**p = 7.2e-13, q = 2.7e-12, t(59) = -9.1, Cohen's dz = -1, SD: 0.074**	**p = 2.8e-27, q = 1.8e-26, t(59) = 19, Cohen's dz = 2.2, SD: 0.075**	**p = 1.1e-18, q = 5e-18, t(59) = 13, Cohen's dz = 1.4, SD: 0.06**
Within	subject 4	**p = 4.1e-05, q = 7.6e-05, t(59) = -4.4, Cohen's dz = -0.5, SD: 0.11**	**p = 1.1e-12, q = 4e-12, t(59) = 9, Cohen's dz = 1, SD: 0.1**	**p = 3e-07, q = 7.1e-07, t(59) = 5.8, Cohen's dz = 0.65, SD: 0.078**
Mixed-Within	subject 5	**p = 3.7e-26, q = 2e-25, t(59) = -18, Cohen's dz = -2.1, SD: 0.1**	**p = 1.3e-26, q = 7.9e-26, t(59) = 19, Cohen's dz = 2.1, SD: 0.1**	p = 0.22, q = 0.26, t(59) = -1.3, Cohen's dz = -0.14, SD: 0.045
Mixed-Within	subject 6	**p = 7.5e-37, q = 7.2e-36, t(59) = -29, Cohen's dz = -3.3, SD: 0.069**	**p = 1.2e-38, q = 2.9e-37, t(59) = 32, Cohen's dz = 3.5, SD: 0.061**	**p = 0.024, q = 0.036, t(59) = -2.3, Cohen's dz = -0.26, SD: 0.04**
Mixed-Within	subject 7	**p = 3.6e-37, q = 3.9e-36, t(59) = -30, Cohen's dz = -3.3, SD: 0.092**	**p = 7.7e-38, q = 1.2e-36, t(59) = 31, Cohen's dz = 3.4, SD: 0.088**	p = 0.078, q = 0.1, t(59) = -1.8, Cohen's dz = -0.2, SD: 0.039
Mixed-Within	subject 8	**p = 3.1e-37, q = 3.7e-36, t(59) = -30, Cohen's dz = -3.3, SD: 0.076**	**p = 4e-40, q = 1.3e-38, t(59) = 34, Cohen's dz = 3.8, SD: 0.068**	p = 0.58, q = 0.59, t(59) = 0.55, Cohen's dz = 0.062, SD: 0.039
Mixed-Across	subject 9	p = 0.43, q = 0.45, t(59) = -0.79, Cohen's dz = -0.089, SD: 0.052	p = 0.4, q = 0.43, t(59) = -0.85, Cohen's dz = -0.095, SD: 0.042	p = 0.066, q = 0.089, t(59) = -1.9, Cohen's dz = -0.21, SD: 0.041
Mixed-Across	subject 10	p = 0.4, q = 0.43, t(59) = 0.84, Cohen's dz = 0.094, SD: 0.04	**p = 6.8e-4, q = 0.0012, t(59) = 3.6, Cohen's dz = 0.4, SD: 0.035**	**p = 5e-4, q = 8.6e-4, t(59) = 3.7, Cohen's dz = 0.41, SD: 0.043**
Mixed-Across	subject 11	p = 0.45, q = 0.46, t(59) = 0.77, Cohen's dz = 0.086, SD: 0.043	p = 0.046, q = 0.063, t(59) = -2, Cohen's dz = -0.23, SD: 0.043	p = 0.24, q = 0.28, t(59) = -1.2, Cohen's dz = -0.13, SD: 0.046
Mixed-Across	subject 12	p = 0.37, q = 0.4, t(59) = 0.91, Cohen's dz = 0.1, SD: 0.042	**p = 0.0077, q = 0.012, t(59) = -2.8, Cohen's dz = -0.31, SD: 0.041**	p = 0.1, q = 0.13, t(59) = -1.6, Cohen's dz = -0.18, SD: 0.046
Across	subject 13	p = 0.094, q = 0.12, t(179) = 1.7, Cohen's dz = 0.12, SD: 0.04	**p = 0.00011, q = 2.1e-04, t(179) = 3.9, Cohen's dz = 0.28, SD: 0.039**	**p = 4.6e-07, q = 9.9e-07, t(179) = 5.2, Cohen's dz = 0.37, SD: 0.043**
Across	subject 14	**p = 0.026, q = 0.038, t(179) = 2.2, Cohen's dz = 0.16, SD: 0.055**	**p = 1.7e-14, q = 6.7e-14, t(179) = -8.4, Cohen's dz = -0.59, SD: 0.044**	**p = 1.5e-07, q = 3.5e-07, t(179) = -5.5, Cohen's dz = -0.39, SD: 0.044**
Across	subject 15	**p = 6.5e-06, q = 1.3e-05, t(179) = 4.6, Cohen's dz = 0.33, SD: 0.043**	**p = 1.5e-08, q = 3.9e-08, t(179) = -5.9, Cohen's dz = -0.42, SD: 0.04**	p = 0.26, q = 0.3, t(179) = -1.1, Cohen's dz = -0.08, SD: 0.034
Across	subject 16	**p = 6.5e-11, q = 2.1e-10, t(179) = -7, Cohen's dz = -0.49, SD: 0.05**	p = 0.12, q = 0.16, t(179) = -1.5, Cohen's dz = -0.11, SD: 0.053	**p = 7.3e-09, q = 2e-08, t(179) = -6.1, Cohen's dz = -0.43, SD: 0.071**

S1		Pre&Cue vs. Post&Cue	Post&Cue vs. Pre&Post	Pre&Cue vs. Pre&Post
Within	subject 1	p = 0.29, q = 0.33, t(59) = 1.1, Cohen's dz = 0.12, SD: 0.1	**p = 4.9e-11, q = 1.6e-10, t(59) = 8, Cohen's dz = 0.9, SD: 0.069**	**p = 3.6e-07, q = 8.3e-07, t(59) = 5.7, Cohen's dz = 0.64, SD: 0.12**
Within	subject 2	**p = 2.6e-10, q = 8.1e-10, t(59) = -7.6, Cohen's dz = -0.85, SD: 0.075**	**p = 1.2e-16, q = 5.1e-16, t(59) = 11, Cohen's dz = 1.3, SD: 0.073**	**p = 0.0017, q = 0.0028, t(59) = 3.3, Cohen's dz = 0.37, SD: 0.08**
Within	subject 3	**p = 8.5e-10, q = 2.5e-09, t(59) = -7.3, Cohen's dz = -0.82, SD: 0.099**	**p = 3.7e-26, q = 2e-25, t(59) = 18, Cohen's dz = 2.1, SD: 0.09**	**p = 6.5e-20, q = 3.1e-19, t(59) = 14, Cohen's dz = 1.5, SD: 0.068**
Within	subject 4	**p = 1.9e-09, q = 5.2e-09, t(59) = -7.1, Cohen's dz = -0.79, SD: 0.079**	**p = 2.1e-20, q = 1.1e-19, t(59) = 14, Cohen's dz = 1.6, SD: 0.077**	**p = 3.9e-12, q = 1.3e-11, t(59) = 8.7, Cohen's dz = 0.97, SD: 0.06**
Mixed-Within	subject 5	**p = 9.1e-28, q = 6.2e-27, t(59) = -20, Cohen's dz = -2.2, SD: 0.095**	**p = 2.1e-28, q = 1.6e-27, t(59) = 20, Cohen's dz = 2.3, SD: 0.089**	p = 0.15, q = 0.18, t(59) = -1.5, Cohen's dz = -0.16, SD: 0.035
Mixed-Within	subject 6	**p = 2.2e-44, q = 2.1e-42, t(59) = -40, Cohen's dz = -4.5, SD: 0.057**	**p = 2.4e-43, q = 1.1e-41, t(59) = 38, Cohen's dz = 4.3, SD: 0.055**	**p = 3.7e-07, q = 8.3e-07, t(59) = -5.7, Cohen's dz = -0.64, SD: 0.026**
Mixed-Within	subject 7	**p = 1.1e-37, q = 1.4e-36, t(59) = -30, Cohen's dz = -3.4, SD: 0.079**	**p = 3.3e-38, q = 6.4e-37, t(59) = 31, Cohen's dz = 3.5, SD: 0.075**	**p = 0.005, q = 0.0082, t(59) = -2.9, Cohen's dz = -0.33, SD: 0.026**
Mixed-Within	subject 8	**p = 3.5e-30, q = 2.8e-29, t(59) = -22, Cohen's dz = -2.5, SD: 0.096**	**p = 2.7e-30, q = 2.4e-29, t(59) = 22, Cohen's dz = 2.5, SD: 0.094**	p = 0.23, q = 0.28, t(59) = -1.2, Cohen's dz = -0.13, SD: 0.038
Mixed-Across	subject 9	p = 0.7, q = 0.7, t(59) = 0.39, Cohen's dz = 0.044, SD: 0.033	**p = 0.013, q = 0.02, t(59) = 2.6, Cohen's dz = 0.29, SD: 0.036**	**p = 0.01, q = 0.016, t(59) = 2.7, Cohen's dz = 0.3, SD: 0.039**
Mixed-Across	subject 10	p = 0.35, q = 0.38, t(59) = 0.94, Cohen's dz = 0.11, SD: 0.037	p = 0.099, q = 0.13, t(59) = -1.7, Cohen's dz = -0.19, SD: 0.034	p = 0.58, q = 0.59, t(59) = -0.56, Cohen's dz = -0.063, SD: 0.038
Mixed-Across	subject 8	p = 0.32, q = 0.36, t(59) = -1, Cohen's dz = -0.11, SD: 0.043	p = 0.26, q = 0.3, t(59) = -1.1, Cohen's dz = -0.13, SD: 0.045	p = 0.043, q = 0.059, t(59) = -2.1, Cohen's dz = -0.23, SD: 0.045
Mixed-Across	subject 9	**p = 0.016, q = 0.025, t(59) = 2.5, Cohen's dz = 0.28, SD: 0.04**	**p = 2.1e-08, q = 5.4e-08, t(59) = -6.5, Cohen's dz = -0.72, SD: 0.037**	**p = 4e-04, q = 7.1e-04, t(59) = -3.8, Cohen's dz = -0.42, SD: 0.038**
Across	subject 10	**p = 1.2e-05, q = 2.3e-05, t(179) = 4.5, Cohen's dz = 0.32, SD: 0.038**	**p = 1.7e-05, q = 3.3e-05, t(179) = 4.4, Cohen's dz = 0.31, SD: 0.034**	**p = 4.3e-17, q = 1.9e-16, t(179) = 9.3, Cohen's dz = 0.66, SD: 0.034**
Across	subject 11	**p = 1e-09, q = 3e-09, t(179) = 6.4, Cohen's dz = 0.46, SD: 0.035**	**p = 7.3e-06, q = 1.5e-05, t(179) = -4.6, Cohen's dz = -0.33, SD: 0.033**	**p = 0.033, q = 0.047, t(179) = 2.2, Cohen's dz = 0.15, SD: 0.034**
Across	subject 12	**p = 0.008, q = 0.013, t(179) = 2.7, Cohen's dz = 0.19, SD: 0.032**	p = 0.34, q = 0.38, t(179) = 0.95, Cohen's dz = 0.067, SD: 0.031	**p = 9.9e-07, q = 2.1e-06, t(179) = 5.1, Cohen's dz = 0.36, SD: 0.023**
Across	subject 16	**p = 0.0012, q = 0.002, t(179) = 3.3, Cohen's dz = 0.23, SD: 0.041**	**p = 2.2e-06, q = 4.6e-06, t(179) = -4.9, Cohen's dz = -0.35, SD: 0.039**	p = 0.15, q = 0.18, t(179) = -1.5, Cohen's dz = -0.1, SD: 0.039

Supplementary Table S3: Single-subject statistical tests for corrected mean representational similarity of within-image comparisons

	V1	Pre&Cue vs. Post&Cue	Post&Cue vs. Pre&Post	Pre&Cue vs. Pre&Post
Within	subject 1	p = 0.85, q = 0.98, t(19) = -0.2,<br>Cohen's dz = -0.044, SD: 0.061	**p = 1.9e-04, q = 0.0026, t(19) = -4.6,<br>Cohen's dz = -1, SD: 0.06**	**p = 7.9e-05, q = 0.0019, t(19) = -5,<br>Cohen's dz = -1.1, SD: 0.058**
	subject 2	p = 0.12, q = 0.4, t(19) = 1.6,<br>Cohen's dz = 0.37, SD: 0.088	p = 0.36, q = 0.72, t(19) = -0.94,<br>Cohen's dz = -0.21, SD: 0.077	p = 0.2, q = 0.53, t(19) = 1.3,<br>Cohen's dz = 0.3, SD: 0.054
	subject 3	p = 0.9, q = 0.99, t(19) = 0.13,<br>Cohen's dz = 0.028, SD: 0.041	**p = 0.0052, q = 0.046, t(19) = -3.2,<br>Cohen's dz = -0.71, SD: 0.058**	p = 0.0081, q = 0.06, t(19) = -3,<br>Cohen's dz = -0.66, SD: 0.06
	subject 4	p = 0.72, q = 0.97, t(19) = -0.36,<br>Cohen's dz = -0.081, SD: 0.053	**p = 1.3e-04, q = 0.0025, t(19) = -4.8,<br>Cohen's dz = -1.1, SD: 0.066**	**p = 1.4e-05, q = 6.8e-04, t(19) = -5.8,<br>Cohen's dz = -1.3, SD: 0.058**
Mixed-Within	subject 5	p = 0.36, q = 0.72, t(19) = -0.94,<br>Cohen's dz = -0.21, SD: 0.075	p = 0.013, q = 0.083, t(19) = -2.7,<br>Cohen's dz = -0.61, SD: 0.073	**p = 4.5e-08, q = 4.3e-06, t(19) = -8.7,<br>Cohen's dz = -2, SD: 0.031**
	subject 6	**p = 1.9e-04, q = 0.0026, t(19) = -4.6,<br>Cohen's dz = -1, SD: 0.039**	p = 0.85, q = 0.98, t(19) = 0.2,<br>Cohen's dz = 0.044, SD: 0.059	**p = 7.5e-05, q = 0.0019, t(19) = -5,<br>Cohen's dz = -1.1, SD: 0.034**
	subject 7	p = 0.012, q = 0.082, t(19) = -2.8,<br>Cohen's dz = -0.62, SD: 0.052	p = 0.23, q = 0.59, t(19) = 1.2,<br>Cohen's dz = 0.27, SD: 0.064	p = 0.051, q = 0.23, t(19) = -2.1,<br>Cohen's dz = -0.47, SD: 0.031
	subject 8	p = 0.07, q = 0.29, t(19) = -1.9,<br>Cohen's dz = -0.43, SD: 0.05	p = 1, q = 1, t(19) = -0.0011,<br>Cohen's dz = -0.00025, SD: 0.047	p = 0.019, q = 0.11, t(19) = -2.6,<br>Cohen's dz = -0.57, SD: 0.038
Mixed-Across	subject 9	p = 0.51, q = 0.85, t(19) = -0.67,<br>Cohen's dz = -0.15, SD: 0.035	p = 0.046, q = 0.22, t(19) = -2.1,<br>Cohen's dz = -0.48, SD: 0.04	**p = 0.0058, q = 0.046, t(19) = -3.1,<br>Cohen's dz = -0.69, SD: 0.035**
	subject 10	p = 0.54, q = 0.85, t(19) = -0.62,<br>Cohen's dz = -0.14, SD: 0.036	p = 0.21, q = 0.55, t(19) = -1.3,<br>Cohen's dz = -0.29, SD: 0.042	p = 0.053, q = 0.23, t(19) = -2.1,<br>Cohen's dz = -0.46, SD: 0.037
	subject 11	p = 0.28, q = 0.6, t(19) = -1.1,<br>Cohen's dz = -0.25, SD: 0.047	p = 0.083, q = 0.33, t(19) = -1.8,<br>Cohen's dz = -0.41, SD: 0.039	**p = 0.0023, q = 0.022, t(19) = -3.5,<br>Cohen's dz = -0.79, SD: 0.035**
	subject 12	p = 0.17, q = 0.48, t(19) = -1.4,<br>Cohen's dz = -0.32, SD: 0.03	**p = 0.0022, q = 0.022, t(19) = -3.5,<br>Cohen's dz = -0.79, SD: 0.034**	**p = 4.5e-04, q = 0.0054, t(19) = -4.2,<br>Cohen's dz = -0.95, SD: 0.038**
Across	subject 13	p = 0.54, q = 0.85, t(19) = -0.63,<br>Cohen's dz = -0.14, SD: 0.037	p = 0.26, q = 0.6, t(19) = -1.2,<br>Cohen's dz = -0.26, SD: 0.036	p = 0.16, q = 0.48, t(19) = -1.5,<br>Cohen's dz = -0.32, SD: 0.045
	subject 14	p = 0.24, q = 0.59, t(19) = -1.2,<br>Cohen's dz = -0.27, SD: 0.042	p = 0.25, q = 0.59, t(19) = -1.2,<br>Cohen's dz = -0.27, SD: 0.042	p = 0.031, q = 0.15, t(19) = -2.3,<br>Cohen's dz = -0.52, SD: 0.043
	subject 15	p = 0.38, q = 0.72, t(19) = -0.9,<br>Cohen's dz = -0.2, SD: 0.041	p = 0.1, q = 0.37, t(19) = -1.7,<br>Cohen's dz = -0.38, SD: 0.03	p = 0.018, q = 0.11, t(19) = -2.6,<br>Cohen's dz = -0.58, SD: 0.035
	subject 16	p = 0.28, q = 0.6, t(19) = 1.1,<br>Cohen's dz = 0.25, SD: 0.038	p = 0.17, q = 0.48, t(19) = -1.4,<br>Cohen's dz = -0.32, SD: 0.05	p = 0.58, q = 0.87, t(19) = -0.56,<br>Cohen's dz = -0.13, SD: 0.053

	S1	Pre&Cue vs. Post&Cue	Post&Cue vs. Pre&Post	Pre&Cue vs. Pre&Post
Within	subject 1	p = 0.49, q = 0.84, t(19) = -0.7,<br>Cohen's dz = -0.16, SD: 0.052	p = 0.49, q = 0.84, t(19) = -0.71,<br>Cohen's dz = -0.16, SD: 0.08	p = 0.3, q = 0.65, t(19) = -1.1,<br>Cohen's dz = -0.24, SD: 0.088
	subject 2	p = 0.96, q = 1, t(19) = -0.047,<br>Cohen's dz = -0.011, SD: 0.056	p = 0.74, q = 0.97, t(19) = 0.34,<br>Cohen's dz = 0.076, SD: 0.052	p = 0.78, q = 0.97, t(19) = 0.29,<br>Cohen's dz = 0.065, SD: 0.052
	subject 3	p = 0.87, q = 0.98, t(19) = -0.17,<br>Cohen's dz = -0.038, SD: 0.069	p = 0.78, q = 0.97, t(19) = -0.28,<br>Cohen's dz = -0.063, SD: 0.063	p = 0.58, q = 0.87, t(19) = -0.56,<br>Cohen's dz = -0.12, SD: 0.053
	subject 4	p = 0.43, q = 0.8, t(19) = -0.8,<br>Cohen's dz = -0.18, SD: 0.046	p = 0.38, q = 0.72, t(19) = 0.9,<br>Cohen's dz = 0.2, SD: 0.047	p = 0.89, q = 0.99, t(19) = 0.13,<br>Cohen's dz = 0.03, SD: 0.041
Mixed-Within	subject 5	p = 0.93, q = 0.99, t(19) = 0.093,<br>Cohen's dz = 0.021, SD: 0.091	p = 0.92, q = 0.99, t(19) = 0.1,<br>Cohen's dz = 0.022, SD: 0.091	p = 0.55, q = 0.85, t(19) = 0.61,<br>Cohen's dz = 0.14, SD: 0.029
	subject 6	p = 0.86, q = 0.98, t(19) = 0.18,<br>Cohen's dz = 0.041, SD: 0.052	p = 0.98, q = 1, t(19) = -0.025,<br>Cohen's dz = -0.0056, SD: 0.062	p = 0.75, q = 0.97, t(19) = 0.33,<br>Cohen's dz = 0.073, SD: 0.024
	subject 7	p = 0.16, q = 0.48, t(19) = -1.4,<br>Cohen's dz = -0.32, SD: 0.059	p = 0.38, q = 0.72, t(19) = 0.89,<br>Cohen's dz = 0.2, SD: 0.06	p = 0.16, q = 0.48, t(19) = -1.5,<br>Cohen's dz = -0.33, SD: 0.021
	subject 8	p = 0.097, q = 0.37, t(19) = -1.7,<br>Cohen's dz = -0.39, SD: 0.057	p = 0.26, q = 0.6, t(19) = 1.2,<br>Cohen's dz = 0.26, SD: 0.063	p = 0.47, q = 0.84, t(19) = -0.73,<br>Cohen's dz = -0.16, SD: 0.034
Mixed-Across	subject 9	p = 1, q = 1, t(19) = -0.0034,<br>Cohen's dz = -7.5e-04, SD: 0.024	p = 0.83, q = 0.98, t(19) = -0.22,<br>Cohen's dz = -0.049, SD: 0.033	p = 0.8, q = 0.97, t(19) = -0.26,<br>Cohen's dz = -0.058, SD: 0.028
	subject 10	p = 0.11, q = 0.4, t(19) = -1.7,<br>Cohen's dz = -0.37, SD: 0.029	p = 0.31, q = 0.65, t(19) = 1,<br>Cohen's dz = 0.23, SD: 0.024	p = 0.47, q = 0.84, t(19) = -0.74,<br>Cohen's dz = -0.17, SD: 0.031
	subject 8	p = 0.53, q = 0.85, t(19) = 0.63,<br>Cohen's dz = 0.14, SD: 0.033	p = 0.2, q = 0.53, t(19) = -1.3,<br>Cohen's dz = -0.3, SD: 0.024	p = 0.73, q = 0.97, t(19) = -0.35,<br>Cohen's dz = -0.079, SD: 0.033
	subject 9	p = 0.67, q = 0.97, t(19) = -0.43,<br>Cohen's dz = -0.096, SD: 0.037	p = 0.99, q = 1, t(19) = -0.017,<br>Cohen's dz = -0.0037, SD: 0.035	p = 0.69, q = 0.97, t(19) = -0.41,<br>Cohen's dz = -0.092, SD: 0.04
Across	subject 10	p = 0.024, q = 0.13, t(19) = 2.5,<br>Cohen's dz = 0.55, SD: 0.022	p = 0.14, q = 0.47, t(19) = -1.5,<br>Cohen's dz = -0.34, SD: 0.029	p = 0.73, q = 0.97, t(19) = 0.35,<br>Cohen's dz = 0.079, SD: 0.025
	subject 11	p = 0.96, q = 1, t(19) = 0.055,<br>Cohen's dz = 0.012, SD: 0.035	p = 0.73, q = 0.97, t(19) = 0.35,<br>Cohen's dz = 0.077, SD: 0.036	p = 0.74, q = 0.97, t(19) = 0.34,<br>Cohen's dz = 0.076, SD: 0.04
	subject 12	p = 0.77, q = 0.97, t(19) = -0.29,<br>Cohen's dz = -0.065, SD: 0.032	p = 0.91, q = 0.99, t(19) = 0.11,<br>Cohen's dz = 0.024, SD: 0.033	p = 0.82, q = 0.98, t(19) = -0.23,<br>Cohen's dz = -0.05, SD: 0.025
	subject 16	p = 0.55, q = 0.85, t(19) = -0.62,<br>Cohen's dz = -0.14, SD: 0.046	p = 0.78, q = 0.97, t(19) = 0.28,<br>Cohen's dz = 0.062, SD: 0.029	p = 0.59, q = 0.87, t(19) = -0.55,<br>Cohen's dz = -0.12, SD: 0.037
